# Translation elongation factor 2 depletion by siRNA in mouse liver leads to mTOR-independent translational upregulation of ribosomal protein genes

**DOI:** 10.1038/s41598-020-72399-4

**Published:** 2020-09-23

**Authors:** Maxim V. Gerashchenko, Mikhail V. Nesterchuk, Elena M. Smekalova, Joao A. Paulo, Piotr S. Kowalski, Kseniya A. Akulich, Roman Bogorad, Sergey E. Dmitriev, Steven Gygi, Timofei Zatsepin, Daniel G. Anderson, Vadim N. Gladyshev, Victor E. Koteliansky

**Affiliations:** 1 Division of Genetics, Department of Medicine, Brigham and Women’s Hospital, Harvard Medical School, Boston, MA 02115 USA; 2 Skolkovo Institute of Science and Technology, Skolkovo, Moscow Region Russia; 3 David H. Koch Institute for Integrative Cancer Research, Massachusetts Institute of Technology, Cambridge, MA 02142 USA; 4 Department of Cell Biology, Harvard Medical School, Boston, MA 02115 USA; 5 Belozersky Institute of Physico-Chemical Biology, Moscow State University, Moscow, 119992 Russia; 6 Department of Chemistry, Lomonosov Moscow State University, Moscow, Russia

**Keywords:** Translation, RNAi, Inhibitory RNA techniques, RNAi therapy

## Abstract

Due to breakthroughs in RNAi and genome editing methods in the past decade, it is now easier than ever to study fine details of protein synthesis in animal models. However, most of our understanding of translation comes from unicellular organisms and cultured mammalian cells. In this study, we demonstrate the feasibility of perturbing protein synthesis in a mouse liver by targeting translation elongation factor 2 (eEF2) with RNAi. We were able to achieve over 90% knockdown efficacy and maintain it for 2 weeks effectively slowing down the rate of translation elongation. As the total protein yield declined, both proteomics and ribosome profiling assays showed robust translational upregulation of ribosomal proteins relative to other proteins. Although all these genes bear the TOP regulatory motif, the branch of the mTOR pathway responsible for translation regulation was not activated. Paradoxically, coordinated translational upregulation of ribosomal proteins only occurred in the liver but not in murine cell culture. Thus, the upregulation of ribosomal transcripts likely occurred via passive mTOR-independent mechanisms. Impaired elongation sequesters ribosomes on mRNA and creates a shortage of free ribosomes. This leads to preferential translation of transcripts with high initiation rates such as ribosomal proteins. Furthermore, severe eEF2 shortage reduces the negative impact of positively charged amino acids frequent in ribosomal proteins on ribosome progression.

## Introduction

Protein synthesis is a very intricate process that requires dozens of protein and RNA molecules to work in a highly coordinated manner. It is regulated both at the transcriptional level in terms of availability of mRNA, and at the translational level in terms of the number of active ribosomes, translational efficiency of mRNA species, rate of elongation, and frequency of initiation and reinitiation events. Since translation consumes the lion’s share of resources and represents a heavy burden on the energy balance in a typical eukaryotic cell^1,2^, evolutionarily conserved mechanisms have been developed to keep translation in-check. One of the central regulatory pathways is the mechanistic Target of Rapamycin (mTOR). In mammals, mTOR kinase serves as a catalytic subunit of two complexes, mTORC1 and mTORC2. mTOR complexes are regulatory hubs where many environment and nutrient-sensing pathways converge. Each complex controls a diverse although partially overlapping set of signaling and metabolic pathways which appears to be involved in nearly every aspect of cell physiology^3,4^. Relevant to this study, mTORC1 is well documented to regulate translation through downstream effectors such as S6 kinase, 4E-BP1, and LARP1^3,5^. Although inhibition of mTORC1 leads to a decrease in cap-dependent translation in general, the primary target of regulation is the translation of a selected pool of mRNAs bearing oligopyrimidine TOP motif at the 5′ end^6–8^. TOP motif-containing genes are almost exclusively comprised of ribosomal proteins, elongation, and initiation factors. Therefore, inhibition of mTORC1 by small molecule inhibitors^9^, deficiency of amino acids^10,11^, or lack of the growth hormone^12,13^ and insulin^14^ results in translational repression of the ribosome machinery, which in turn, slows down translation of other mRNAs. The molecular mechanism of these interactions has been intensively studied over the past three decades (reviewed in^3,5^). A thorough experimental work was performed to unwind the entire mTOR signaling network from the extracellular stimuli down to the exact mechanisms of translational control. Briefly, rich nutrient and growth-promoting conditions activate the kinase activity of mTORC1, which in turn, phosphorylates 4E-BP1 and LARP1 proteins enabling TOP motif binding by eIF4G and eIF4E initiation factors and enhancement of translation efficiency of ribosomal genes^7,15–17^.

Research on aging is one of the areas where the mTOR function is particularly intriguing. It is commonly accepted that many lifespan-extending interventions share translation as a common downregulated pathway^18–20^. The decrease in global translation and enhanced translation of specific mRNA subsets are the common outcomes of these interventions^21^. Similar conclusions were drawn from studies done on a broad repertoire of species—from yeast to mammals^22–26^. However, at the most fundamental level, it remains unclear to what extent the global reduction in mRNA translation contributes to the increased lifespan. Furthermore, mTOR participates in many cellular processes, not only in translation regulation, making it hard to discriminate the input of any given pathway.

In this study, we were driven by the idea of perturbing protein synthesis uniformly in a specific tissue in vivo, with minimal involvement of major signaling pathways. What would be a cellular response to the translation repression from within the cell as opposed to from the outside? Is it possible to entirely bypass the mTOR pathway? To do this, we employed an in vivo siRNA treatment to suppress translation elongation in mouse liver. Elongation relies on two essential protein factors with non-overlapping function—eEF1 and eEF2^27,28^. Unlike eEF1 and many initiation factors, eEF2 is encoded by a single gene which makes it the ideal candidate for downregulation by RNA interference^29^. We have targeted eEF2 to cause the deficiency and slow down protein synthesis in the liver. This treatment was expected to affect protein synthesis without discrimination since all ribosomes rely on the same elongation factor to catalyze a translocation event. We also conducted a set of histopathology tests, proteomics, transcriptomics, and ribosome profiling analyses. *Eef2* knockdown in vivo but not in vitro led to the strongly upregulated synthesis of ribosomal proteins highlighting cellular attempts to compensate for poor protein production. However, the entire branch of the mTORC1 pathway responsible for translation control including mTOR, S6K, and 4E-BP1 was unperturbed.

We have also compared the results with our recent study on *Eif3* subunits knockdown in the mouse liver, thus implementing different strategies of impairing translation in vivo—at the initiation stage and at the elongation stage.

## Results

### *Eef2* knockdown in cell culture negatively impacts cell cycle and has no effect on mTOR pathway

**Figure 1:**
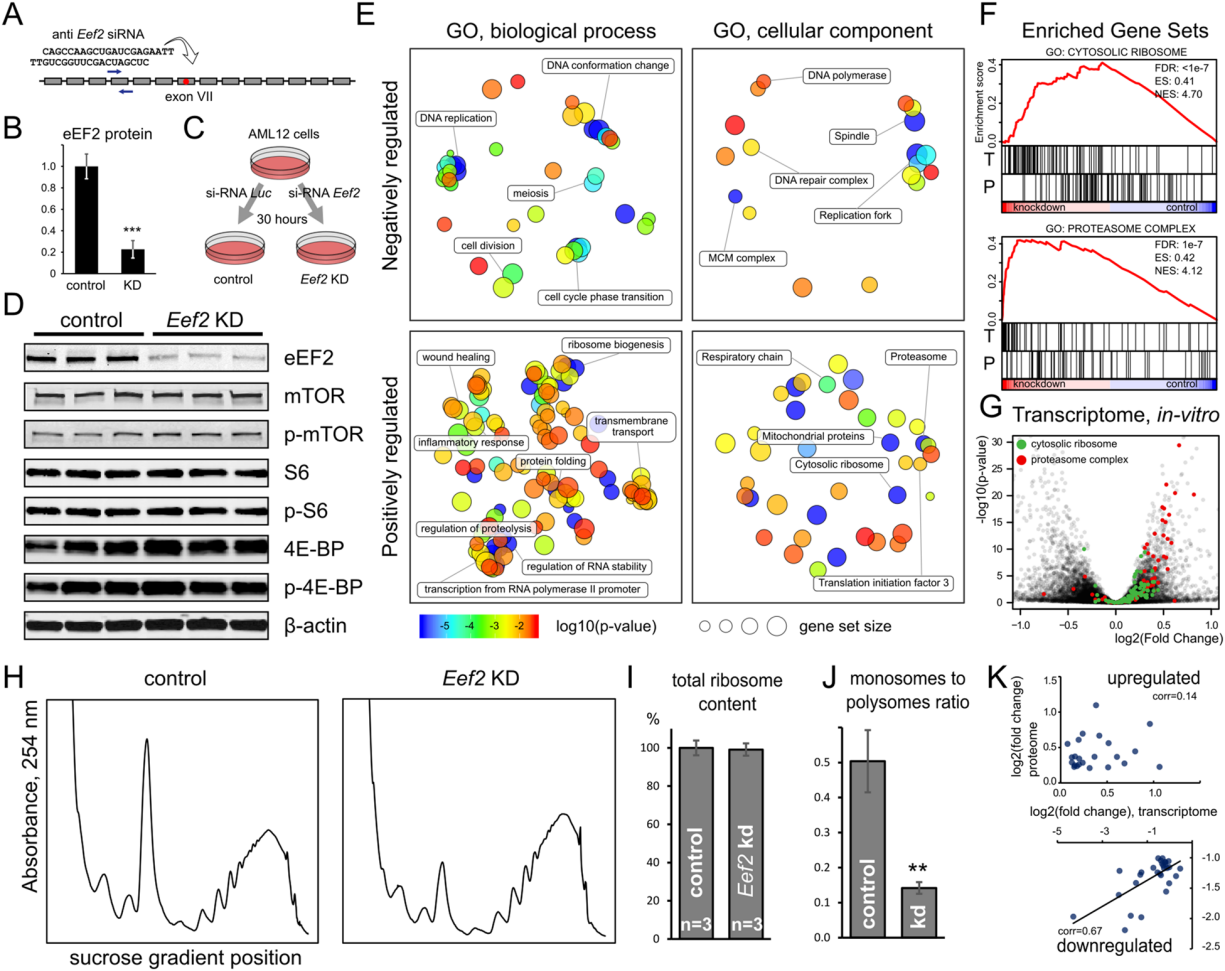
*Eef2* knockdown in AML12 cell line. (**A**) Anti *Eef2* siRNA sequence and the target region. (**B**) eEF2 protein depletion in AML12 cells after 2 days with siRNA. Error bars show the standard error of the mean (**C**) Experimental design scheme. (**D**) Western blotting analysis of cell lysates probed against eEF2, beta-actin and members of the mTOR regulatory pathway. Each condition is represented by three independent biological replicates. (**E**) Gene Ontology clustering and visualization by REVIGO. Children categories are collapsed and hidden within their parents whenever possible. Related categories are clustered together based on the semantic similarity. Input GO term lists were obtained from GSEA pre-ranked analysis of the transcriptional response to the *Eef2* knockdown. (**F**) Examples of Cytosolic Ribosome and Proteasome Complex gene sets derived from the GSEA analysis of transcriptomic (T) and proteomic (P) data. Red line shows the cumulative enrichment score for the given gene set and vertical stripes represent individual genes contained within the set. (**G**) Volcano plot of all genes taken from the control vs knockdown comparison. (**H**) Sucrose gradient profiles of ribosomes from cell lysates. (**I**) Total ribosomal content is not changed by *Eef2* knockdown (**J**) *Eef2* knockdown converts monosomes to polysomes. (**K**) Comparison between gene expression changes measured by mRNA-seq and protein mass-spectrometry. Top upregulated genes and equal number of downregulated genes from proteomics data set were compared with corresponding fold-changes from the transcriptomic data set. Pearson correlation is included.

12 modified siRNAs targeting elongation factor 2 (*Eef2*) were designed, synthesized, and tested in cell culture. Similar to our previous studies^30,31^, siRNAs were tested in HEPA 1–6 (Mouse hepatic carcinoma) and AML12 (Mouse hepatocytes) cell lines to estimate the efficacy of siRNA for mRNA knockdown in hepatocytes in vivo. The most potent siRNA with IC50 1.3 pM at mRNA level was selected for further experiments (Fig. 1A, Supplementary Fig. S1). After 30–48 h of siRNA treatment, the amount of eEF2 protein was reduced down to 20% as measured by Western blotting (Fig. 1B–D). Subsequent polysome profiling analysis revealed significant rearrangement of ribosomes from monosomal to polysomal fractions while the total amount of ribosomes remained constant (Fig. 1H–J). These observations were confirmed by the large-scale quantitative proteomics of control and *Eef2* knockdown AML12 cells. According to the proteomics data, the amount of eEF2 protein decreased fourfold upon 48 h of treatment (Supplementary Fig. S2). At the same time, the relative abundance of ribosomal proteins did not change. Thus, we could achieve impaired translation elongation without activating ribosome biogenesis. We have also verified the extent of translation slow down by measuring protein production for 24 h using luciferase reporters delivered to cells via transfection of in vitro transcribed capped RNA. The difference between the control and knockdown cells was 4–7-fold after 4 h, depending on the reporter and the full time-lapse as presented in Fig. S3.

A cell can control the number of ribosomes by increasing translation efficiency of transcripts coding ribosomal proteins via the mTOR pathway^3,7^. We measured the most common members of the mTOR translation regulation pathway including mTOR, S6, and 4E-BP1 proteins by Western blotting. Neither expression level nor the phosphorylation status was changed for any of these targets in response to *Eef2* knockdown (Fig. 1D). Protein expression levels were additionally validated by proteomics data (Supplementary Fig. S2). To better characterize the impact of the knockdown, we screened AML12 cells with the custom panel of ~ 400 antibodies targeting various regulatory proteins and corresponding phosphorylation sites^32^. The screen confirmed Western blotting-based conclusions, and we found no evidence of other mTOR pathway components such as Raptor, Rictor, P70 S6K, p90RSK, to be affected by the *Eef2* knockdown (Supplementary Fig. S4, Supplementary Data 1).

We characterized the transcriptional response to *Eef2* knockdown in AML12 cell line by sequencing mRNA isolated from control cells and cells treated with si-*Eef2* for 2 days (n = 4 in each group). ~ 14,000 genes were detectable in both experimental groups. We performed gene set enrichment analysis (GSEA) using signed p-values as the ranking metric. Enriched gene ontology categories (GO) were clustered together based on the semantic similarity as shown in Fig. 1E. Among molecular functions, almost all downregulated genes were related to cell division. Examples include histone and chromatin binding, DNA helicase activity, etc. Similarly, biological processes GO categories are represented by DNA replication, meiosis, cell division and cell cycle phase transition (Fig. 1E). It may be concluded that cell division is most dispensable when translation is impaired. On the other hand, among genes upregulated by *Eef2* knockdown, gene sets related to translation and proteolysis stood out. Examples include ribosome biogenesis, regulation of proteolysis, protein folding, rRNA binding, and other related categories. To illustrate the most meaningful cases, we selected two well-defined gene lists: Cytosolic Ribosome and Proteasome Complex. Both are at the top of statistically significant gene sets (Fig. 1F). It should be noted that, while the response was robust, the extent of the genes expression changes contained in these sets was moderate. It does not exceed 50% for ribosomal and twofold for proteasomal proteins (Fig. 1G).

We performed quantitative protein mass spectrometry of the same cell line to determine how closely transcription correlates with translation considering the fact that we directly target translation (Supplementary Data 2). Comparison of enriched gene sets with transcriptomic data showed similar GO categories downregulated: DNA replication, repair, meiosis, cell division and cell cycle (Supplementary Fig. S5). Consistent with polysome gradient profiles, cytosolic ribosome proteins were not enriched in the knockdown. Proteasomal genes, while statistically significant, were not as strongly enriched in the knockdown phenotype compared to transcriptome data and the Spearman correlation of GSEA ranks between transcriptomic and proteomic data was only moderate 0.4.

We took a closer look at the top differentially expressed genes in response to *Eef2* knockdown from proteomic and transcriptomic data sets. For the top downregulated genes there was a correlation 0.67 between expression fold-change values measured by mass-spec and mRNA-seq. On the other hand, there was no correlation between proteomics and mRNA-seq for upregulated genes (Fig. 1K).

In conclusion, we demonstrated the efficacy of siRNA to decrease the abundance of elongation factor 2 while preserving cell viability for 48 h. At the same time, we did not detect strong feedback loops that might activate ribosome biogenesis or translation of ribosomal protein genes. Also, the mTOR pathway did not appear to be activated by eEF2 depletion. This encouraged us to examine the consequence of eEF2 depletion in the mouse model.

### *Eef2* knockdown in mouse liver increases ribosomal content and triggers a decline in liver function

**Figure 2:**
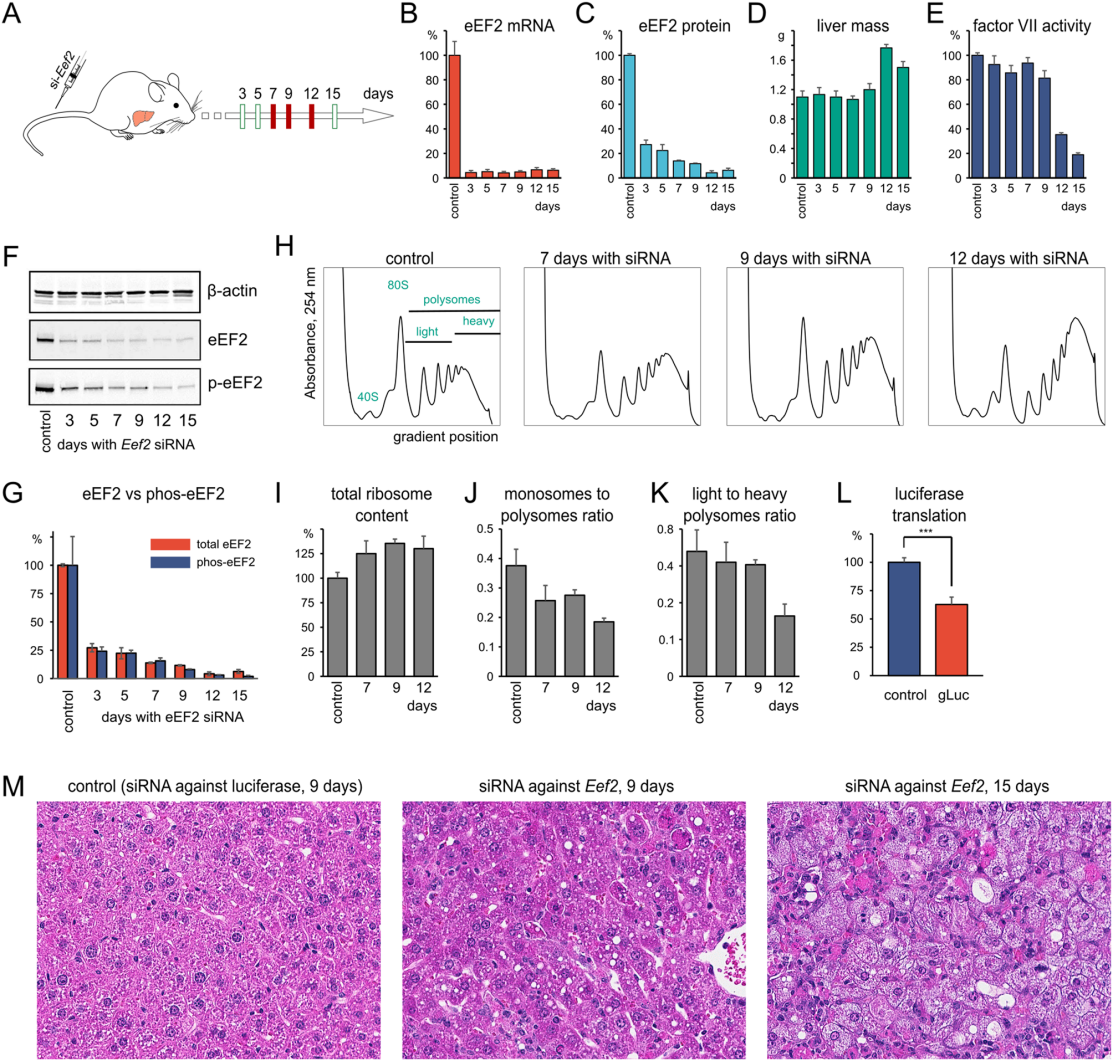
Consequences of *Eef2* knockdown in mouse liver. (**A**) Experimental design, sample collection days, and injection schedule. Red marks days when samples were analyzed with omics approaches. (**B**) *Eef2* mRNA depletion by siRNA in a mouse liver. (**C**) Decrease in eEF2 protein content in response to siRNA. (**D**) The decrease in eEF2 causes hepatomegaly after 9 days of treatment with siRNA. (**E**) Factor VII activity in the serum decreases sharply after 9 days of treatment with siRNA. (**F**) Western blots with antibodies against total eEF2 and phosphorylated at threonine-56. (**G**) Phosphorylation status of eEF2 does not change during the knockdown progression. (**H**) Representative sucrose gradient polysomal profiles of mice livers. (**I**) Ribosome content increases with time. (**J**) The pool of free ribosomes gets depleted as more of them engage in elongation and get stalled on mRNA due to lack of the elongation factor. (**K**) Ribosomes rearrange from light to heavy (> 4 ribosomes per mRNA) fraction of polysomes. (**L**) Excretable luciferase reporter knock-in in the liver of mice with suppressed *Eef2*. Serum luciferase activity is measured 6 h after luciferase transcript delivery. (**M**) Hematoxylin and eosin staining of mouse liver sections. Lipid droplets start accumulating in response to the knockdown. Note: error bars on every bar plot show standard deviation and every time point has at least 3 animals.

We aimed to silence the expression of *Eef2* in the mouse liver using lipid nanoparticle (LNP) formulations of siRNA optimized for hepatic delivery^30,33,34^. The same siRNA as in in vitro experiment was formulated into LNP and injected via tail vein into mice at the 0.5 mg/kg dose. Control mice were injected with the formulation containing luciferase siRNA at the same dose (Fig. 2A). Single injection led to ~ 95% *Eef2* mRNA reduction in the liver after 3 days (Fig. 2B). We performed siRNA LNP injections twice a week to maintain constant suppression of *Eef2*. Knockdown at the protein level was evaluated by Western blot analysis over the course of a 15-day treatment with siRNA LNP. eEF2 protein level decreased rapidly by 70% within the first 3 days and reached the maximum knockdown of approximately 95% after 12 days of treatment (Fig. 2C,F).

To monitor knockdown progression, we measured various parameters of liver function, including factor VII activity and serum biochemistry. Factor VII is synthesized and secreted by the liver; its activity sharply decreased after nine days of the siRNA treatment indicating a failure of the liver to perform a normal function (Fig. 2E). Mice with *Eef2* knockdown also exhibited reduced concentration of alkaline phosphatase—the protein synthesized and secreted by the liver (Supplementary Table S1). Moreover, the liver mass increased by 20% after day 9 but neither signs of inflammation nor fibrosis were observed (Fig. 2D). Concordantly, serum markers of liver damage, such as alanine and aspartate aminotransferases showed a manifold increase in the signal (Supplementary Table S1). Histopathology has further revealed the extent of liver damage inflicted by impaired translation. Hematoxylin and eosin staining of liver sections detected formation of lipid droplets and protein inclusions on day 9 in full agreement with dysregulation of hepatic metabolism. The number and the size of droplets increased further and by the day 15 one could see hepatocyte membrane damage (Fig. 2M, Supplementary Figs. S6, S7). Lipid droplets depositions was further confirmed by Oil Red staining (Fig. S8). In agreement, serum concentrations of cholesterol and triglycerides decreased manifold (Supplementary Table S1).

To validate the extent of the translation repression, we used in vivo knock-in of the mRNA encoding Gaussia luciferase reporter. mRNA was formulated into LNPs and intravenously injected in mice held on the anti *Eef2* siRNA for 9 days. The reporter was delivered to the liver and directly translated into the naturally excreted luciferase^31^. The luciferase activity in the serum, measured 6 h after injection, was twofold lower than the luciferase activity from control mice (Fig. 2L).

eEF2 is regulated by threonine-56 phosphorylation which makes it less active^35,36^. We detected no compensatory activation of eEF2 and its phosphorylation state did not change with the progression of the knockdown. Since eEF2 depletion is expected to influence protein synthesis in general, we assessed the translation state of hepatic cells by analyzing polysome profiles in a sucrose gradient (Fig. 2H). The total ribosomal content increased by 20–25% per tissue weight indicating attempts to compensate for impaired translation by increasing the number of ribosomes (Fig. 2I). The pool of free ribosomes was depleted as the ratio of monosomes to polysomes decreases more than twofold (Fig. 2J). Similarly, ribosomes within polysomes rearranged from light to heavy (> 4 ribosomes per mRNA molecule) fraction (Fig. 2K). These observations suggest that elongating ribosomes accumulate on mRNA competing for sparse eEF2 molecules.

### Proteome analysis identifies ribosomal proteins, initiation and elongation factors as the most upregulated targets

**Figure 3:**
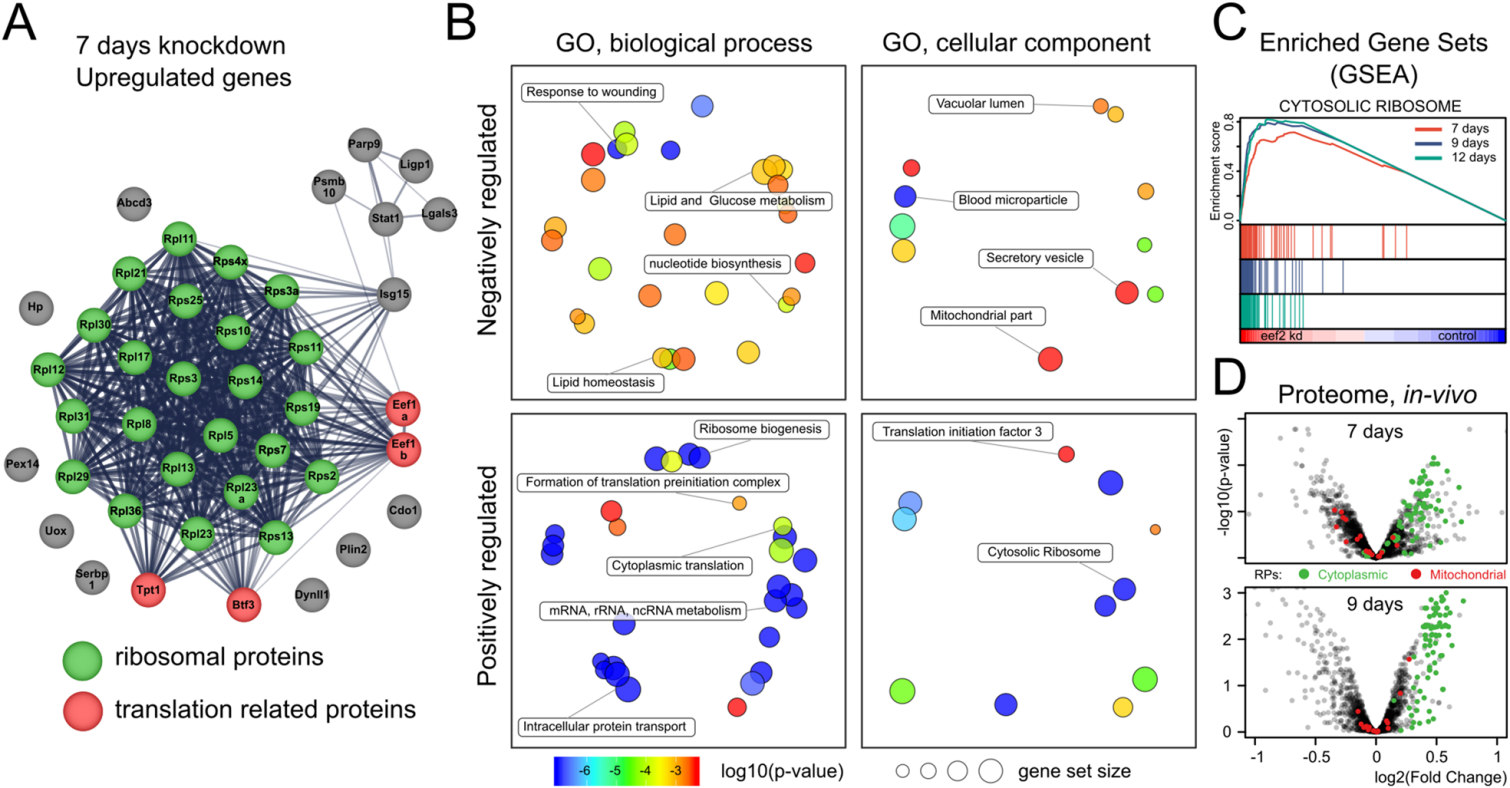
Mass spec proteomics of *Eef2* knockdown in mouse liver. (**A**) Enrichment analysis of upregulated proteins. Ribosomal proteins are colored in green, proteins related to translation regulation—in red. (**B**) Gene ontology enrichment of up- and down-regulated proteins. (**C**) Proteins comprising cytosolic ribosome display a positive enrichment in the knockdown phenotype with the enrichment score gradually increasing with the knockdown progression. (**D**) Cytosolic proteins are the top hits in both statistical significance and effect size.

We employed tandem mass tag LC–MS/MS protein mass spectrometry to characterize liver protein content of *Eef2* knockdown mice. Seven, nine, and twelve days post-injection time points were chosen based on the pattern of the factor VII activity and transcriptomic analysis of the corresponding animals. Luciferase siRNA injected mice were used as a control group. Three replicates of control mice and three replicates of siRNA treated mice were compared with each other. The grand total of 1996 proteins were included in the analysis after passing quality filters and at least two unique peptides per protein requirement (Supplementary Data 3). The differential expression analysis was conducted with *limma* package in R. The first time point (7 days) yielded less than 30 differentially regulated proteins indicating a very early response to the *Eef2* knockdown, consistent with the decline of serum factor VII (Fig. 3A). At the next time point (day 9) a total of 34 proteins were found upregulated and 18 downregulated (Benjamini–Hochberg corrected p-value < 0.05). eEF2 served as a positive internal control as it appeared at the top of the downregulated proteins both by the fold change and the p-value. As the number of differentially regulated proteins was low and the majority of proteins did not exceed 1.5-fold change (Fig. 3D), we did not impose any additional thresholds and cut-offs. Strikingly, among the 34 upregulated proteins 24 were ribosomal proteins from both small and large subunits as can be seen in STRING visualization (Supplementary Fig. S9). During the progression of knockdown from 9 to 12 days, the cluster of upregulated ribosomal proteins became even larger. We also detected two other eukaryotic elongation factors eEF1a1 and eEF1b among upregulated proteins. Two other interesting translation-related proteins appeared upregulated—BTF3 and LARP1. BTF3 is the general transcription inhibitor, but it has an important secondary function as a component of the nascent polypeptide associated complex (NAC). LARP1 functions downstream of mTORC1 and regulates translation of 5′ TOP motif-containing mRNAs. Taken together, these observations indicate that the subset of transcripts encoding translation machinery components is the main target of the knockdown. Most of the transcripts of ribosomal proteins, elongation and some initiation factors contain TOP motifs, therefore the tight upregulation of the entire cluster of ribosomal proteins can be attributed to mTOR, 4E-BP1 and LARP1 pathway. Furthermore, as mRNAs encoding the mitochondrial ribosome proteins lack TOP motif, they were not upregulated or repressed by the translation elongation factor knockdown (Fig. 3D). To further investigate the effect of *Eef2* knockdown, we performed GSEA on the same set of proteomics data. Genes were pre-ranked using the signed logarithm of the p-value provided as an output from the differential gene expression analysis. GSEA clearly supported upregulation of genes involved in translation, ribosomal biogenesis, mRNA and ncRNA metabolism. Pretty much every gene encoding the cytosolic ribosome was enriched in the knockdown phenotype based on the GSEA analysis (Fig. 3C,D). There was a very strong enrichment of related GO categories (Fig. 3B). Gene sets enriched among downregulated genes were less conclusive with the prevalence of secretory pathways and exocytosis categories, which is not surprising given the general decline in liver function and progressing signs of factor VII depletion in the serum (Fig. 2E, Supplementary Table S1).

Overall, the proteomics study indicated the liver attempts to upregulate translation machinery to compensate for the impaired protein synthesis in eEF2 deficient animals. A moderate degree of upregulation (expressed as a fold change) for most of the statistically significant ribosomal proteins in proteomic data is consistent with a polysome profiling analysis which detected ~ 25% increase in the total number of ribosomes. Considering that all ribosomal proteins, elongation, and many initiation factors are already one of the most abundant proteins in the cell makes it is hardly possible to increase their quantities any further. The statistical significance and the extent of enrichment of the TOP motif-containing mRNAs were remarkably robust (Fig. 3C,D).

### *Eef2* knockdown enhances translation of mRNAs with the TOP motif

Regulation of the TOP containing mRNAs should manifest itself at the translational level. To confirm it, we performed mRNA-seq (poly(A) selected transcriptomics) and Ribo-seq (ribosome profiling). Same samples of mouse liver were analyzed as in the mass-spec proteomics study. Therefore, transcriptomic, ribosome profiling and proteomic data could be matched within individual animals.

**Figure 4:**
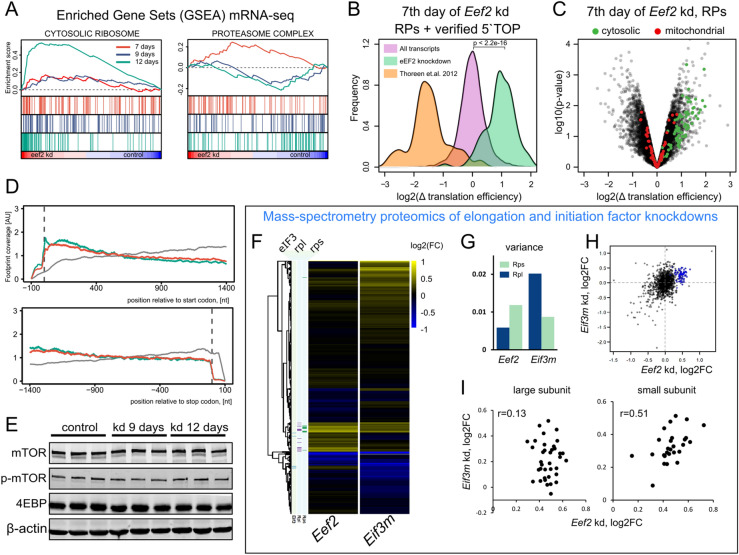
Transcriptome and Ribosome Profiling analysis of *Eef2* knockdown in the mouse liver. (**A**) Gene set enrichment analysis of mRNA-seq with two representative gene ontology groups. (**B**) Translation efficiency change of transcripts containing TOP motif and comparison to the same genes from Thoreen et al. (2012). (**C**) Translation efficiency change on the 7th day of *Eef2* knockdown, comparison between cytosolic and mitochondrial ribosomes. (**D**) Normalized ribosome footprint coverage over coding sequences. (**E**) Western blot analysis of mTOR and phospho-mTOR in the liver of control and *Eef2* knockdown mice shows no activation of mTOR pathway. (**F**) Quantitative proteomics of *Eef2* and *Eif3m* knockdowns in the mouse liver. Hierarchical clustering of the protein expression fold change values. Protein components of the eIF3 complex, large and small ribosomal subunits are shown to the left of the main heatmap (**G**) Expression of large ribosomal subunit components is tighter regulated during *Eef2* knockdown. (**H**) Comparison of fold changes between *Eef2* and *Eif3m* liver knockdowns. Ribosomal proteins are highlighted in blue. (**I**) *Eef2* and *Eif3m* knockdowns both have increased expression of ribosomal proteins, but the small and the large subunits show discordant regulation with the changes in the small subunit proteins being correlated across knockdowns.

Transcriptome sequencing was performed for the liver samples of 7, 9, and 12 days after the first *Eef2* siRNA-LNP injection and compared to a 9 day mock injection control. We acquired 12 million high-quality paired-end reads per sample on average with good reproducibility between replicates which was enough to detect ~ 12.000 genes. Early days of the knockdown showed no significant changes of the transcripts encoding cytosolic ribosomal proteins (Fig. 4A). Intriguingly, the late time point does demonstrate a strong positive enrichment of the corresponding gene set. It appears that the depletion of eEF2 has a bimodal response when the TOP motif-containing genes are activated at the translation level first and later further boosted transcriptionally. The concept of time-dependent changes in the knockdown progression is further reinforced by the proteasome complex gene set enrichment. It was slightly positively enriched at the 7 day time point and then suppressed in later days (Fig. 4A). Days 7 and 9 of the knockdown showed a reasonably small set of differentially regulated genes provided the twofold cut-off was imposed. On the other hand, the last time point (12 days of siRNA treatment) had many differentially expressed genes and evident liver damage as confirmed by histopathology (Supplementary Figs. S6,S7) and blood biochemistry. As such, the biological relevance of the observed enrichment was not evident.

There was a clear discrepancy between proteomic and transcriptomic sets of differentially expressed genes at the early days of knockdown which implied post-transcriptional regulation. Therefore, we examined the mRNA content of actively translating ribosomes by means of Ribo-seq. Ribo-seq enhances mRNA-seq by surveying actively translating ribosomes and estimating translation efficiency^37,38^. We compared control mice (7 days with anti-*Luc* siRNA) to 7 days of *Eef2* siRNA treatment, three animals in each group. Briefly, crude lysates from mouse livers were subjected to RNase T1 treatment to convert polysomes to monosomes and to get rid of mRNA located outside of translating ribosomes. The ribosomal fraction was purified by ultracentrifugation and short mRNA fragments (footprints) were extracted and sequenced. We acquired 7–9 million uniquely mappable footprints per sample which allowed detection and statistical assessment of approximately 8,500 genes. The data were analyzed in the pipeline similar to the regular mRNA-seq (DESeq2 package in R). According to Ribo-seq footprint coverage, 73 genes were upregulated and 97 downregulated based on the twofold expression change cut-off and the Benjamini–Hochberg corrected p-value less than 0.05. When we examined the upregulated genes, it became evident that by far the largest functional cluster within this data set was represented by 12 protein members of ribosomal subunits (Data tables are available in the GEO repository). The follow-up GSEA analysis further strengthened this observation by revealing a very strong enrichment of GOs “Ribosome biogenesis” and “Ribosome” gene lists towards being overrepresented in *Eef2* knockdown.

Ribo-seq and mRNA-seq data sets can be combined to obtain ribosome occupancy, which is often interpreted as translation efficiency, *i.e.* the measure of how well the transcript is translated compared to other transcripts. Figure 4B-C summarizes the difference in translation efficiency change of ribosomal transcripts. We detected no oddities in the averaged ribosome occupancy over mRNA that could influence the translation efficiency estimation between conditions (Fig. 4D). There was only a minor difference which is the missing peak of the ribosome occupancy over start codon and 5′ UTRs in the knockdown (Fig. 4D). This is the direct consequence of the shortage of ribosomes available for translation initiation. Our study provides convincing evidence of translational upregulation of ribosomal proteins in response to the eEF2 depletion in vivo.

This study relies on the analysis of three large-scale independent data sets: proteomics, transcriptomics, and ribosome profiling. Naturally, if any single data set would have systematic technical issues it might change whether we consider genes to be regulated at the transcriptional or post-transcriptional level. Therefore, it is important to note that the two largest functional clusters of genes downregulated in the transcriptomic dataset were also downregulated in both proteomics and ribosome profiling data sets. Members of the first cluster, chaperones such as *Hspa1, Hspb1, Hsph1, Hspa4l, Hsp90aa1*, were all downregulated by eEF2 depletion in transcriptomic and Ribo-seq experiments. The same holds true for the enzymes involved in acetyl-Coenzyme A and lipid biosynthesis: *Acaa1b, Acacb, Acss2, Acot3, Fasn, Alas1,* and *Me1*. Hence, these two groups of genes were regulated transcriptionally compared to ribosomal protein genes that were regulated translationally.

Despite the difference in the repertoire of translationally activated genes between in vivo and in vitro experiments, neither mTORC1 nor 4E-BP1 phosphorylation status was affected as measured by Western blotting (Fig. 4E). Therefore, the translational activation of ribosomal transcripts in the liver does not occur via mTOR-dependent signaling.

## Discussion

In this study, we explored if a highly abundant essential gene can be knocked down by siRNA technology. The knockdown manifested differently in vitro (ALM12 murine cell line) and in vivo (liver of adult mice). *Eef2* knockdown in the liver led to an increase in the relative abundance of ribosomal proteins due to translational upregulation. On the other hand, in the cell culture there was no change in the number of ribosomes. To clarify, the *Eef2* knockdown suppresses the net translation and the yield of every protein is reduced. However, ribosomal proteins are suppressed less and appear upregulated relative to other proteins based on differential expression analysis. At the same time, both in vivo and in vitro knockdowns shut down expression of genes required for cell proliferation at the level of transcription.

**Figure 5:**
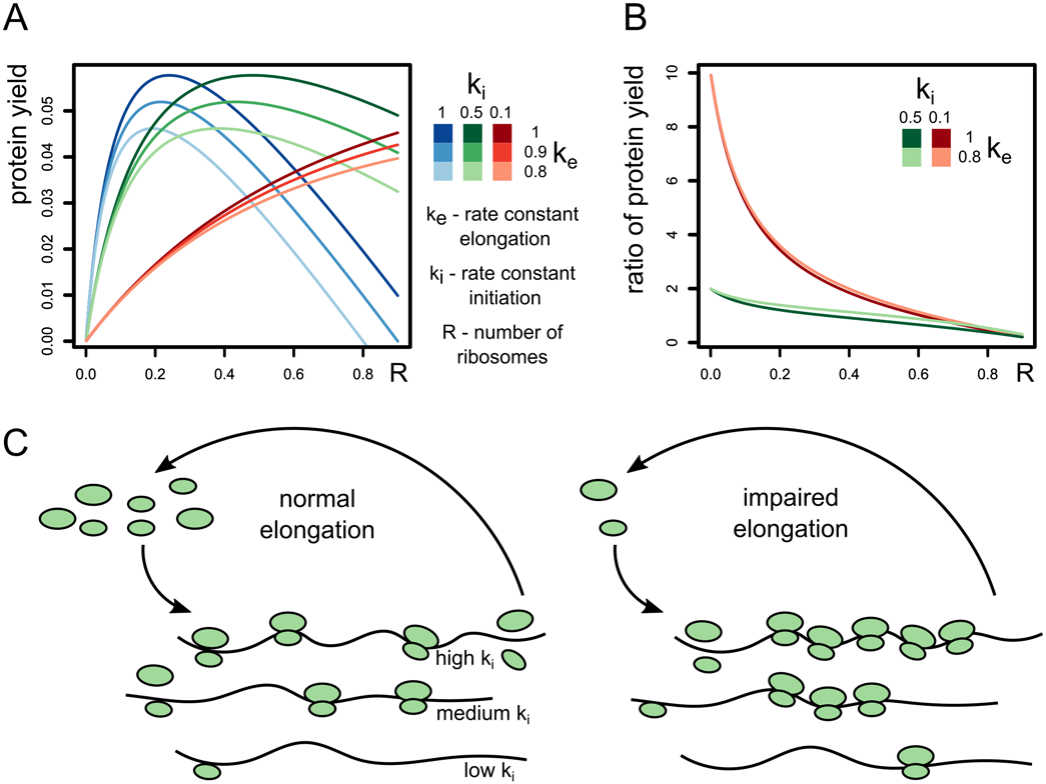
Slow elongation rate selectively increases translation of transcripts with higher initiation rates. (**A**) Protein yield of transcripts with 3 different initiation and elongation constants depends on availability of free ribosomal subunits. (**B**) The ratio of protein yield between the transcript with initiation constant [ki] = 1 and transcripts with lower k_i_. The difference is larger at lower R. Additional changes to elongation [k_e_] have no effect on the ratio. (**C**) Model of translation at normal conditions and at *Eef2* knockdown. Impaired elongation sequesters ribosomes on mRNA. Shortage of free ribosomes leads to the preferential translation of transcripts with high initiation rate.

Considering the high representation of ribosomal proteins at the top translationally upregulated genes, we suspected involvement of the mTOR pathway. However, further experiments clearly showed no signs of mTOR activation. Upon further investigation, we considered another mechanism of post-transcriptional control which senses the elongation rate rather than nutrient availability or upstream signaling. According to hypotheses and computational simulations, transcript-specific translation can be achieved by changing the ratio between available ribosomes and mRNA molecules^39–41^. This fact follows from the analysis of the following equation:

$$Q=mR{k}_{i}\left[1-\frac{L}{\frac{{k}_{e}}{{k}_{i}R}+L-1}\right]$$

where *Q* is the protein yield, *R* is the concentration of free ribosomes, *k*_*i*_ and *k*_*e*_ are rate constants for initiation and elongation respectively, *m* is the concentration of mRNA, L is the length of mRNA covered by a single ribosome, and the term in brackets is the probability that the initiation codon is not covered by a ribosome and available for initiation. Figure 5A,B analyses this equation for several model transcripts with varying *k*_*i*_, *k*_*e*_, and *R*. Our observations can be explained by the passive nature of such regulation. Impaired elongation sequesters ribosomes on the mRNA creating a shortage of free unbound ribosome subunits. At the same time, the amount of initiation factors still remains high. Transcripts engage in competition for ribosomes at these conditions and those with higher initiation rate win. If ribosomal transcripts are somehow more competitive for ribosomes, they would be able to increase the relative yield of corresponding proteins. Transcripts encoding ribosomal proteins indeed have high initiation rates. They have short unstructured 5` UTRs and the pyrimidine-rich motif at the 5′ terminus. Therefore, ribosomal transcripts should be even more efficient when there is shortage of ribosomes capable of initiation (Fig. 5C). Our study provides direct experimental evidence in support of this theoretical model. Moreover, elongation rate is slow on transcripts encoding ribosomal proteins, partially counteracting the impact of the high initiation rate on the final protein yield^42^. This effect is attributed to the interaction between negatively charged exit tunnel of the ribosome and positively charged amino acids of the nascent polypeptide chain^43^. When we halted elongation by creating the deficiency of eEF2, the ribosome decoding time on any given codon became longer. Therefore, the negative impact of positively charged amino acids decreased proportionally as the time waiting for the available eEF2 molecule prior to translocation increased but the time required for the act of translocation remained the same. This secondary effect should boost relative translation of ribosomal proteins even further. Perhaps, it makes an even greater impact then the initiation constant in this case.

Studying gene knockdown effects in live animals presents new interesting challenges to the result interpretation. We find it very likely that impaired translation elongation is the main contributing factor to the overexpression of ribosomal proteins. There is, however, a possibility that the damage done to the liver is itself a driving force behind ribosomal proteins overproduction. In that case, we would anticipate the mTOR pathway to be involved through the upstream stress signaling. The lack of mTOR activation and minor changes to other cell signaling hubs (Supplementary Fig. S4) further supports our original interpretation.

Slow elongation is expected to cause lack of ribosomal subunits not engaged in translation. It should resemble the scenario of impaired initiation. Serendipitously, we used the same method of siRNA delivery to study the function of the m subunit of translation initiation factor 3 in another study^44^. Deep knockdown of *Eif3m* also resulted in the decrease of translation and liver failure after 2–3 weeks. Thus, we could compare two different strategies of impairing translation in vivo—at the initiation stage and at the elongation stage. Here we compared quantitative liver proteomics from *Eif3m* and *Eef2* knockdowns in mice (Fig. 4F). Ribosomal proteins were upregulated in both instances; however, *Eef2* knockdown clearly showed a stronger effect which could be attributed to the difference in knockdown duration between the two experiments (Fig. 4F,H). Interestingly, *Eef2* knockdown appears to have a much smaller variance in the regulation of large but not small ribosomal subunits when compared to *Eif3m* knockdown (Fig. 4G,I). At the same time, there is no correlation between the fold-changes of proteins from the large ribosome subunit between *Eef2* and *Eif3m* knockdowns. On the contrary, small subunit proteins have the Pearson correlation coefficient of 0.51.

In addition to what we consider as the main drivers responsible for the increase in the content of ribosomal proteins, there are other important considerations that could not be tested by the current experimental design, for instance, protein degradation. We cannot discount the possibility of the ribosomes to last longer without disassembly and subsequent degradation when the translation is impaired. It could contribute to the observed accumulation of ribosomes and even the increased variance between individual proteins. Stoichiometry of ribosomal proteins has to be well maintained in cells due to the large production volume and therefore high energy requirements. It is achieved through various mechanisms at the level of transcription, translation (TOP motif), and degradation^45^. The severely impaired yield of translation may lead to a decrease in protein degradation to preserve the remaining proteins. Overall, stringent mechanisms regulating stoichiometry of ribosomal protein synthesis may become less selective leading to higher variance across ribosomal protein levels.

Interestingly, we observed this phenomenon only in vivo. When *Eef2* knockdown was performed in the dividing murine cell line—there was no sign of translational upregulation of ribosomal proteins. It is reasonable to expect that it takes time for ribosomal proteins to increase their abundance through the aforementioned passive regulation mechanism, especially when the protein synthesis itself is targeted. In our in vivo studies, we collected the first time point at day 7, whereas in vitro sample collection was performed on the second day. On the other hand, in vitro and in vivo systems chosen for this study could have other properties setting them apart. For example, the basal level of ribosomal protein abundance in actively proliferating cultured cells may be higher than in differentiated hepatocytes in the liver, thus making it harder to elevate it further. Looking at the GSEA enrichment patterns of ribosomal genes at the transcription level, response to *Eef2* knockdown in vitro is most similar to the latest days of the knockdown in vivo (Figs. 1F, 4A). It is plausible that, for some reason, the murine cell line skipped the early phases of translational response observed in the liver and went directly to the transcriptional response. The time series of transcriptome changes obtained during our in vivo experiments clearly demonstrates the complexity of changes.

We would like to emphasize the importance and value of RNAi mediated downregulation of various RNA targets in vivo for adult animals. eEF2 is an essential protein and no heterozygous transgenic mice are available. Even more—transgenic animals can adapt to the decrease of eEF2, so one can hardly study the consequences of eEF2 depletion in such model. Translational upregulation of ribosomal proteins observed in our study was not anticipated and would have been undetected, should we perform our experiments exclusively using immortalized cultured cells. According to the literature, inhibition of mTOR by rapamycin decreases translation of TOP motif-containing transcripts and extends lifespan. Essentially, we observed the exact opposite outcome of *Eef2* knockdown on the gene expression as we expected, and the decrease in elongation rate caused the increase in relative abundance of ribosomes. Granted, the knockdown was quite severe, and we were aiming to disrupt normal protein synthesis as much as possible. It remains to be seen what would happen with a more moderate degree of elongation rate slowdown. Concluding, we were able to directly inhibit translation by targeting the elongation rate while completely bypassing the mTOR pathway. This study presents an opportunity for future analyses of the causal role of translation in lifespan control.

## Methods

### Mouse cell lines

siRNA screenings and efficacy tests were done in Hepa 1–6 mouse cell line (Hepatic carcinoma, ATCC CRL-1830). Transcriptional response to *Eef2* knockdown was assessed in AML12 cell line which was established from hepatocytes derived from CD1 mouse, line MT42 and transgenic for human TGF alpha (ATCC CRL-2254). AML12 cells exhibit typical hepatocyte features such as peroxisomes and bile canalicular-like structure as well as high expression of albumin, antitrypsin, and transferrin. In vitro translation assays were performed in NIH/3T3 mouse cell line (ATCC CRL-1658).

### Mouse strain and siRNA delivery strategy

FVB/NCrl female mice were purchased from Charles River Laboratories. All animals received humane care and study protocols were approved by the Committee on Animal Care of MIT. Animals were maintained in a conventional barrier animal facility with a climate-controlled environment on a 12-h light/12-h dark cycle, fed ad libitum with regular rodent chow. Eight weeks old mice received LNP-formulated siRNA at the dose of 0.5 mg/kg by tail vein injection. Animals received two injections per week. Blood samples were collected via submandibular bleeding. Animals were euthanized by carbon dioxide overdose, and tissues samples were collected.

### Tissue samples preparation and histology

For histological analysis, freshly collected tissues were fixed in 4% buffered paraformaldehyde and embedded in paraffin. Five-micrometer-thick sections were stained by hematoxylin–eosin. For total RNA isolation, proteomics and ribosome profiling, tissues were frozen in liquid nitrogen and stored at -80 C.

### Reverse phase protein array (RPPA)

The antibody screening was performed at The University of Texas, MD Anderson Cancer Center, RPPA Core facility using RPPA-Set153 comprising 422 antibodies^32^. Cell lysates were serially diluted (undiluted, 1:2, 1:4, 1:8; 1:16) and arrayed on nitrocellulose-coated slides in an 11 × 11 format to produce sample spots. Sample spots were then probed with antibodies by a tyramide-based signal amplification approach and visualized by DAB colorimetric reaction to produce stained slides. Stained slides were scanned on a Huron TissueScope scanner to produce 16-bit tiff images. Sample spots in tiff images were identified and their densities quantified by Array-Pro Analyzer. Relative protein levels for each sample were determined by interpolating each dilution curve produced from the densities of the 5-dilution sample spots using a "standard curve" (SuperCurve) for each slide (antibody). SuperCurve is constructed by a script written in R. Relative protein levels were expressed in log2 values, normalized for protein loading and transformed to linear values (Supplementary Table S1).

### siRNA screening and LNP formulation

12 siRNAs targeting mouse *Eef2* gene sequence with the lowest off-target potential were designed as described previously^33^. siRNAs were synthesized and chemical modifications (modified bases (2′OMe) and phosphorothioate linkages) were introduced to stabilize siRNA *in-vivo* reducing the off-target potential of the sense strand and minimize immune response (Supplementary Table S3). siRNAs screened in Hepa 1–6 cell line. Cells were transfected with siRNA using Lipofectamine RNAiMAX (Invitrogen). siRNA with the lowest IC50 (determined by western blot and qPCR) was selected for further *in-vitro* and *in-vivo* experiments. Selected siRNA was formulated into lipid nanoparticles. The water phase contained siRNA duplex and ethanol phases with lipids (C12-200, 1,2-distearoyl-sn-glycero-3-phosphocholine (DSPC, Avanti Polar Lipids), cholesterol (Sigma), C14 PEG 2000 (Avanti Polar Lipids) at a 50:10:38.5:1.5 molar ratio) were mixed together in a microfluidic chip device. LNPs were dialyzed overnight against PBS. LNP sizes were measured by dynamic light scattering (ZetaSizer, Malvern Instruments). Mean diameter of the particles prepared for injections was about 90–120 nm.

### Measuring translation rate in vivo

*Gaussia* luciferase (GLuc) mRNAs was synthesized by an in vitro transcription from a DNA template as described previously^31^. Final, purified mRNAs contained a 5′ cap (Cap1), a 5′ UTR consisting of a partial sequence of the cytomegalovirus immediate-early 1 gene, a GLuc coding region, and a 3′ UTR consisting of a partial sequence of the human growth hormone (hGH) gene, and a 3′ polyA tail estimated to be approximately 120 nucleotides long. mRNA was formulated into LNP as previously described. LNP were intravenously injected in mice at the 0.5 mg/kg doze. Blood samples were collected via submandibular bleeding 6 h after the injection. The activity of GLuc in the serum was quantified by BioLux Gaussia Luciferase Assay Kit (NEB).

### Measuring translation rate in vitro

Capped polyadenylated mRNAs for transfection were prepared as described previously^46^. In brief, a T7 promoter-containing, 50A-tailed PCR products were obtained from the plasmids pActin-Fluc^47^ and pCMV-LUC2CP/ARE^48^—a gift from Gideon Dreyfuss, (AddGene #62857), purified by agarose gel electrophoresis, and used as a template for in vitro transcription. RNA synthesis was performed using the RiboMAX kit (Promega), followed by capping by Vaccinia Capping System (NEB). RNA products were purified by LiCl precipitation and checked for integrity by denaturing urea PAGE.

HEK293T cells were grown in DMEM (Gibco) supplemented with 10% FBS (HyClone) in the presence of penicillin and streptomycin (Paneco) in a humidified 5% CO2 atmosphere at 37 °C. At the morning of day 1, cells were trypsinized, counted and transferred either into 96-well plates (white FB/HB Grenier Bio-One LUMITRAC™ #655,074, for luminescence measurement, or a regular transparent one, for microscopic inspection of cell density; both in 75 μl of medium per well), or onto 10-cm dishes (for replating at day 2). During the transfer, the cell suspension was transfected with siRNAs (at final concentration 10 nM), using Lipofectamine RNAiMax and Opti-MEM (ThermoFisher). At the evening of day 2, cells in the 96-well plates were inspected and those with ~ 70% confluency were transfected with the reporter mRNAs. It should be noted that anti-eEF2 siRNA transfected cells were growing a little bit slower, so we prepared serial dilutions of control cells during plating and selected wells with an equal cell count at the time of transfection. For mRNA transfection, per 1 well, 30 ng of mRNA in 20 μl Opti-MEM was mixed with a solution of 0.06 μl of Unifectin-56 (Unifect Group) in 3 μl Opti-MEM, incubated for 15 min, then supplied with 0.4 μl 100 mM D-luciferin (Promega) per well, and added to cells in the LUMITRAC™ plate. During this step, all manipulations were performed as described in the regular FLERT protocol^46^. Real-time luminescence measurements were carried out overnight in the CLARIOstar plate reader (BMG Labtech) equipped with Atmospheric Control Unit to maintain 5% CO2, at 37 °C (light integration time—5 s).

### Western blotting

Samples of powdered liver tissue were homogenized in RIPA buffer with protease inhibitors cocktail (Thermo Fisher Scientific), protein concentrations were measured by BCA assay, 30 µg of total protein samples were run on TGX gradient gels (Bio-Rad), transferred to nitrocellulose membranes and incubated with primary antibodies overnight. Protein bands were visualized by secondary antibodies labelled with infrared fluorophores (IRDye680 or IRDye780). Membranes were scanned on an Odyssey Scanner (LI-COR) and images were processed and quantified with ImageJ. Antibodies used in this study are listed in the Supplementary Table S2. Full-size scans are presented in the Supplementary Fig. S10-11.

### *Eef2* knockdown in AML12 cells

Cells were transfected with LNP formulated siRNA (10 nM). Cells were harvested 30–48 h after transfection and total RNA isolated as in the case of liver tissue samples.

### Polysome profiling

To compare ribosomal content between samples we used a polysome profiling in a sucrose gradient. Ultracentrifuge settings, UV recording, and lysis buffers are the same as in the “Ribosome profiling” section. In cell culture, we normalized input material by the cell count then processed samples together in a single batch in the same ultracentrifuge run. Since counting cells in a frozen liver is not feasible, we weighted frozen liver pieces instead and then selected the pieces closest to 20 mg to ensure the comparable rate of ribosome extraction. After the lysis, lysates were further diluted if necessary, to account for weight differences of the original liver pieces. Number of liver samples exceed the rotor capacity; therefore, they were processed in 3 batches with the same controls present in all of them.

### Ribosome profiling

20–30 mg of frozen liver was used as a starting material. Liver was lysed in a glass-Teflon Dounce homogenizer filled with the ice-cold buffer (20 mM Tris–HCl pH 7.5 at 4 °C, 50 mM KCl, 50 mM NaCl, 5 mM MgCl_2_, 1% Triton X100, 0.2 mg/ml cycloheximide, Roche complete EDTA-free inhibitors). Then it was cleared from debris by 2 min centrifugation at 12,000×*g*. Heparin was added to the supernatant to the final concentration 400 µg/ml. To recover a larger number of intact ribosomes we treated lysates with 400 U/ml of RNase T1 (Epicentre, cat# NT09500K) for 30 min at the room temperature following the guidelines from here^38^. Ribosomes were fractionated by ultracentrifugation in a 10–50% sucrose gradient (20 mM Tris–HCl pH 7.5 at 4 °C, 50 mM KCl, 50 mM NaCl, 10 mM MgCl_2_, 0.2 mg/ml cycloheximide), SW41 rotor, 35.000 rpm, 3 h, 4 °C, Beckman Optima L-20 K centrifuge. After the centrifugation, gradients were passed through a UV detector (Bio-Rad) and the absorption at 254 nm was recorded. The monosome containing fraction was collected in a single tube. The volume of the sample was brought to 50 µl by concentrating it using 100 kDa filters (Amicon Ultra, Millipore). Total RNA content was isolated with Trizol (Ambion) and Direct-Zol miniprep plus kit (Zymo Research) and precipitated by the glycogen-ethanol (1/10 volume of 3 M sodium acetate, 1/100 volume of glycogen, 2.5 volumes of pure ethanol, 1 h incubation at -20 followed by centrifugation). RNA was loaded on a 15% polyacrylamide TBE-urea gel and the band containing ribosomal footprints around 25–32 nucleotides was cut.

### Transcriptome sequencing

Total RNA content was isolated with E.Z.N.A.® Total RNA Kit (Omega Bio-Tek) followed by on-column digestion with RNase-free DNase I. Sample quality was examined by TapeStation2000 (Agilent). Every sample had an RNA integrity number (RIN) higher than 8.5 which indicates high-quality RNA.

### Quantitative PCR

Total RNA was isolated as described above. cDNA was synthetized using the High-Capacity cDNA Reverse Transcription Kit (Thermo Fisher Scientific). Levels of specific mRNAs were measured by qPCR in Roche LightCycler 480. *Gapdh* and *Actb* were used as housekeeping genes for normalization (refer to Table S3 for primer sequences).

### Quantitative proteomics

Powdered liver samples or flash-frozen cell pellets were lysed in 8 M urea, proteins were reduced with dithiothreitol and alkylated with iodoacetamide. Proteins were then digested with modified trypsin and peptides were labeled with TMT 11plex, as described previously^49^. Samples labeled with the different isotopic TMT tags were combined and fractioned via high-pH reverse phase HPLC followed by LC-MS^3^.

### High throughput sequencing and data processing

Ribosome profiling and mRNA-seq libraries were sequenced on the Illumina NextSeq 500 platform at MIT. Detailed pipeline for sequencing reads pre-processing, mapping and differential gene calling can be found in the Supplementary. Custom R scripts used in this study are included as the supplementary files. The NCBI mouse genome build GRCm38.p3 and the *Mus musculus* Annotation Release 105 were used as a reference. When aligning mRNA-seq and ribosome profiling reads for expression and translation efficiency estimation, we used the following strategy. Only full chromosomes were kept and all extra-chromosomal and mitochondrial records removed. Furthermore, only RefSeq and BestRefSeq records were kept in the transcriptome annotation, while Gnomon predictions were discarded along with pseudogenes. Genes with 5′ UTRs shorter than 100 nt were extended up to 100 based on the genomic coordinates.

## Supplementary information

Supplementary file1

Supplementary file2

Supplementary file3

Supplementary file4

## Data Availability

Raw sequencing data files, processed reads-per-gene, differential gene expression tables, and Gene Set Enrichment tables are available at the GEO repository, accession number GSE136091. LC/MS^3^ proteomics results are available as Supplementary Data files on the publisher’s website. Bioinformatical vignettes, analytical pipeline, and scripts required to reproduce this study are available here https://github.com/germaximus/Nesterchuk_2018.
