# Supplementary material for: Translation elongation factor 2 depletion by siRNA in mouse liver leads to mTOR-independent translational upregulation of ribosomal protein genes
Source: Sci Rep. 2020 Sep 23;10:15473. doi: 10.1038/s41598-020-72399-4 (PMC7511953; doi:10.1038/s41598-020-72399-4)
Supplement: Supplementary file 4 — Supplementary file4 [file 41598_2020_72399_MOESM4_ESM.pdf]

Translation elongation factor 2 depletion by siRNA in mouse liver leads to mTOR-independent translational upregulation of ribosomal protein genes

Maxim V. Gerashchenko[1,*,#], Mikhail V. Nesterchuk[2,*], Elena M. Smekalova[3,*], Joao A. Paulo[4], Piotr S. Kowalski[3], Kseniya A. Akulich[5], Roman Bogorad[3], Sergey E. Dmitriev[5], Steven Gygi[4], Timofei Zatsepin[2,6], Daniel G. Anderson[3], Vadim N. Gladyshev[1] and Victor E. Koteliansky[3,#]

* - These authors contributed equally to this work
[1] - Division of Genetics, Department of Medicine, Brigham and Women's Hospital, Harvard Medical School, Boston, MA 02115, USA
[2] - Skolkovo Institute of Science and Technology, Skolkovo, Moscow Region, Russia
[3] - David H. Koch Institute for Integrative Cancer Research, Massachusetts Institute of Technology, Cambridge, MA 02142, USA
[4] - Department of Cell Biology, Harvard Medical School, Boston, MA 02115, USA
[5] - Belozersky Institute of Physico-Chemical Biology, Moscow State University, Moscow 119992, Russia
[6] - Department of Chemistry, Lomonosov Moscow State University, Moscow, Russia
[#] - Correspondence and requests for materials should be addressed to V.K (email: kotelianskiv@gmail.com) and M.V.G. (email: mgerashchenko@bwh.harvard.edu)

a

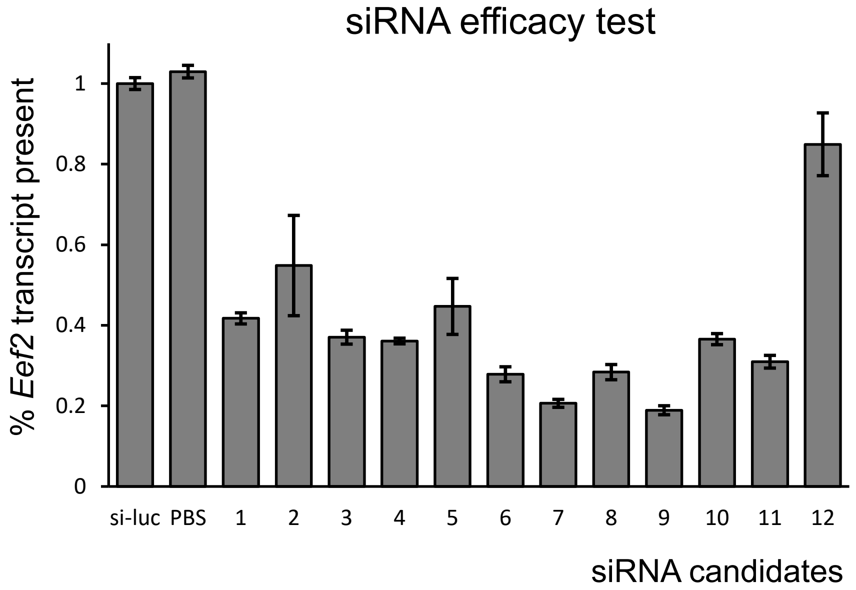

b

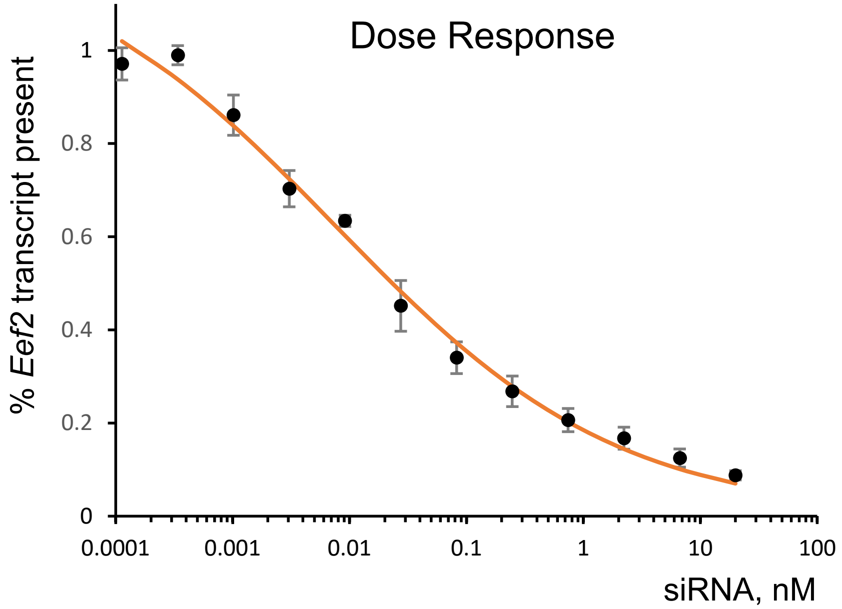

Supplementary figure 1. (**a**) Testing the efficacy of siRNA candidates in the AML12 cell line with qPCR. Phosphate buffer saline (PBS) and LNPs with anti luciferase siRNA were used as negative controls. Each siRNA transfection was done in duplicates. Error bars show the range of measurements. (**b**) Dose-response profile for the most potent candidate.

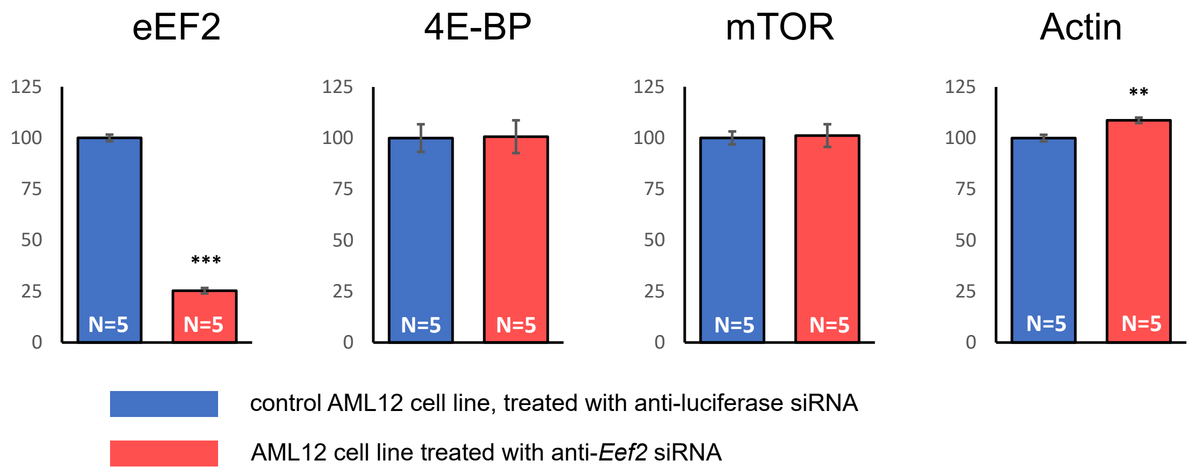

Supplementary figure 2. Protein abundance levels determined by the whole-cell protein mass spectrometry in AML12 cells subjected to 48 hours of *Eef2* knockdown. Proteomics was performed in a quantitative mode with TMT labeling.

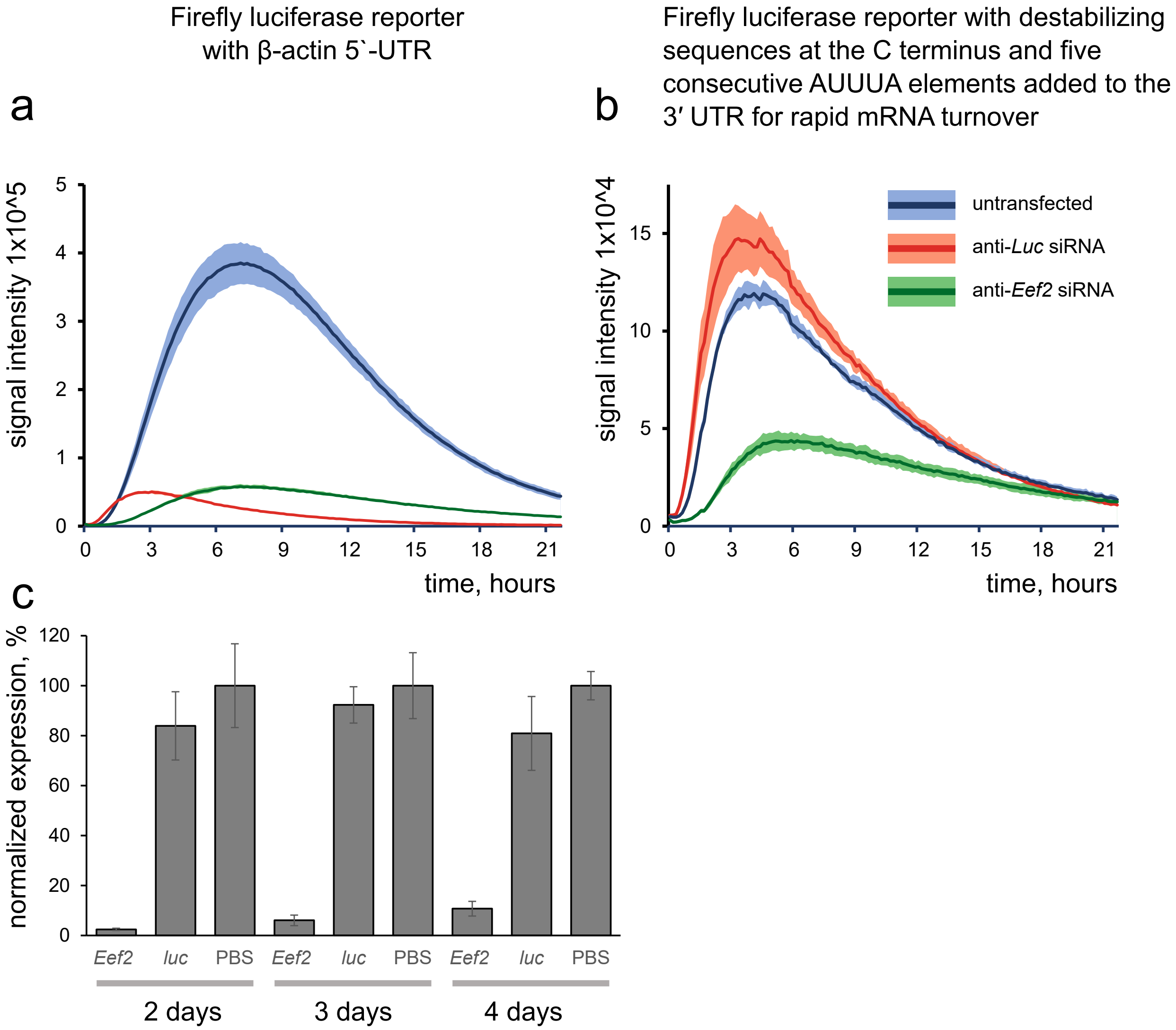

Supplementary figure 3. Protein synthesis yield is reduced by *Eef2* knockdown *in vitro*. NIH/3T3 cells were transfected with siRNA for 2 days, then capped and polyadenylated mRNAs, Actin-Fluc (**a**) or LUC2CP/ARE (**b**) was transfected. Anti-Luc siRNA used as a negative control is designed after Fluc coding region and thus targets Actin-Fluc mRNA but not LUC2CP/ARE mRNA as the latter has a modified luciferase coding region with a degron and mRNA destabilizing element. n=5 in each group in (a) and 3 in (b). Shades represent standard deviation. (**c**) qPCR measurements of *Eef2* mRNA levels in the same conditions as the luciferase reporter assay, normalized to *Gapdh* housekeeping gene. Error bars show standard deviation.

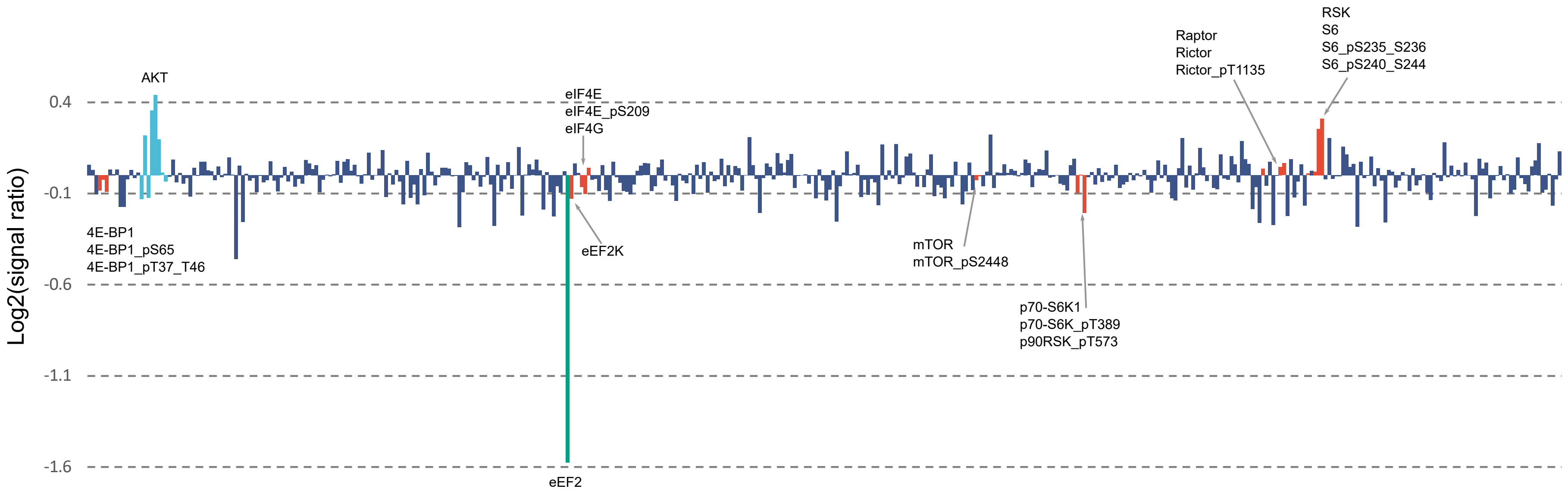

Supplementary figure 4. Reverse phase protein array. 422 antibodies probing target proteins and their phosphorylation state. Comparison is made between control AML12 cells and *Eef2* knockdown. Red bars highlight mTOR and translation related proteins. Green bar corresponds to eEF2 and light blue bars to AKT1-2. Experiment was conducted in triplicates (3 controls and 3 knockdown replicates). Raw values and identities of every antibody target can be found in the Supplementary Table S1.

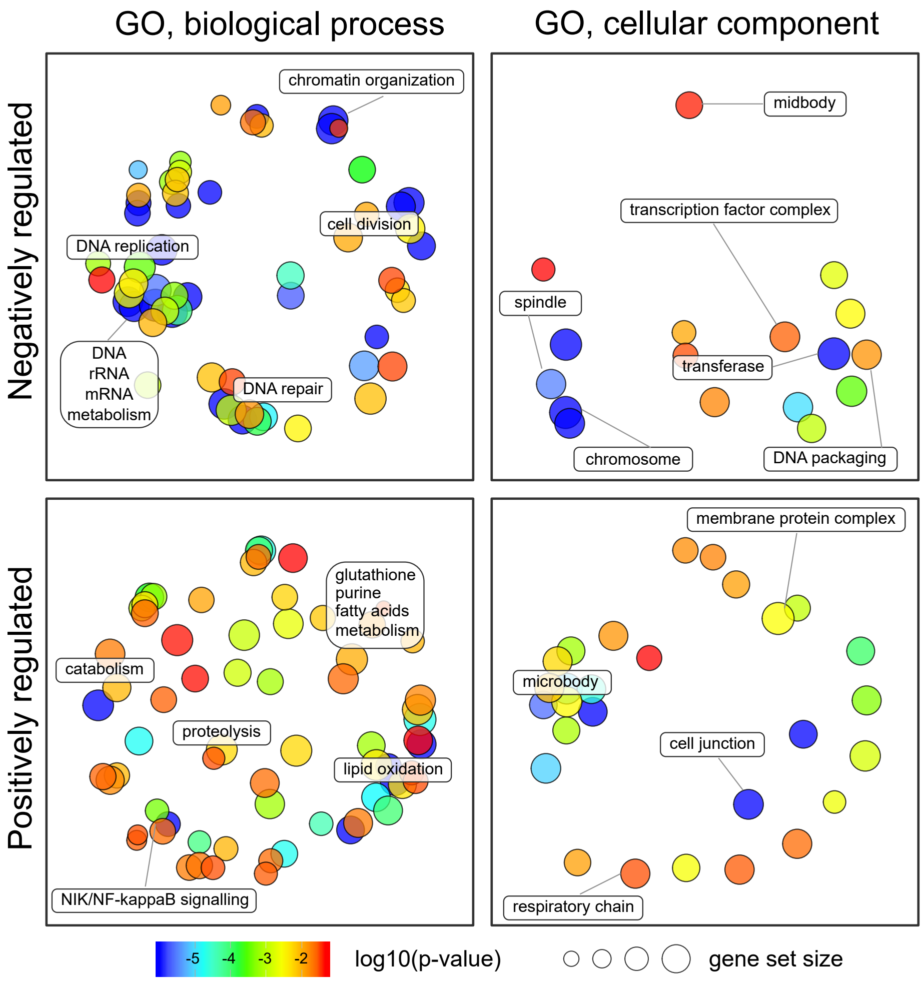

Supplementary figure 5. Proteomics analysis of the *Eef2* knockdown in the AML12 cell line. Gene Ontology clustering and visualization by REVIGO. Children categories are collapsed and hidden within their parents whenever possible. Related categories are clustered together based on the semantic similarity. Input GO term lists were obtained from the GSEA pre-ranked analysis of the transcriptional response to the *Eef2* knockdown.

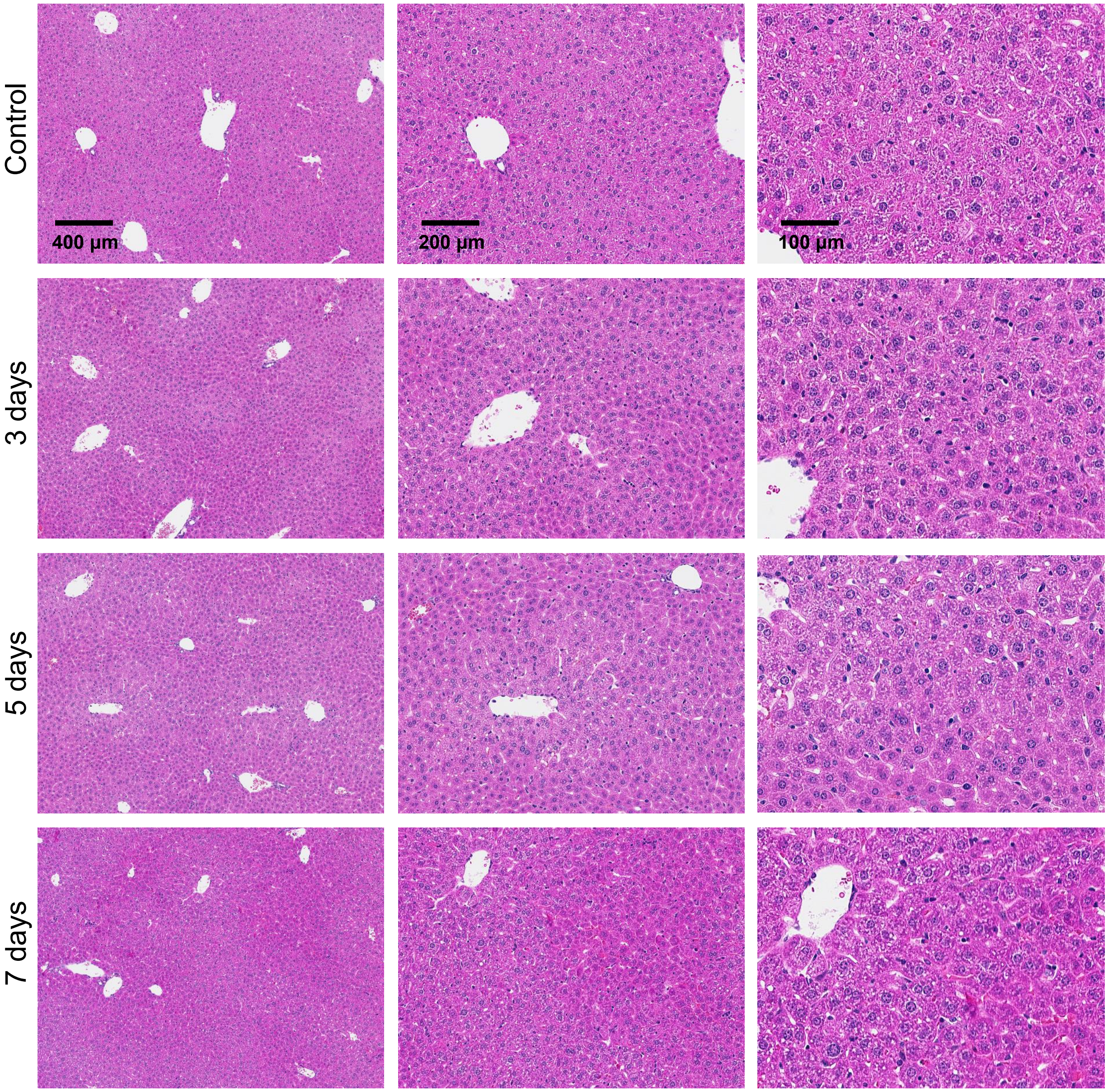

Supplementary figure 6. H&E staining of liver sections. Part I. Mice were injected with anti *Eef2* siRNA and sacrificed on specified days.

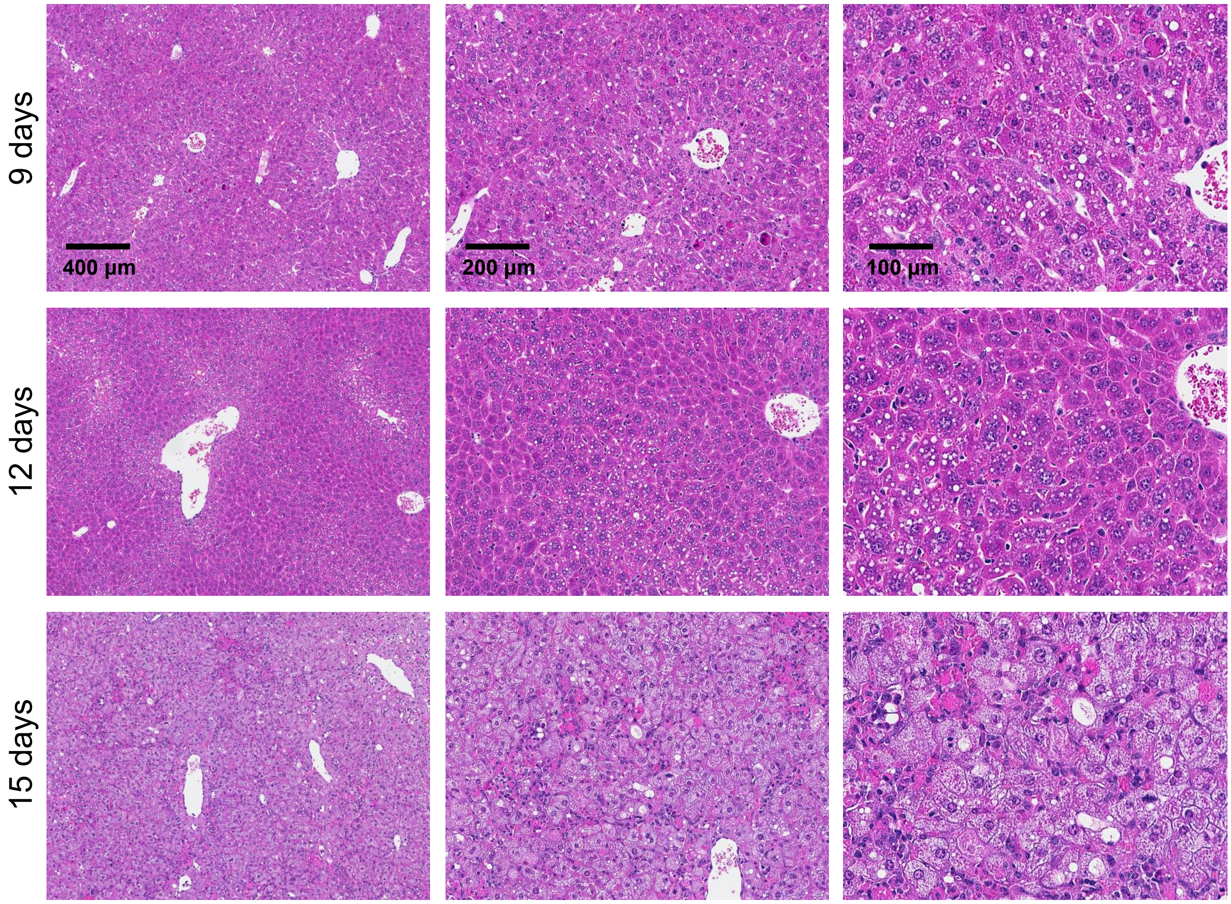

Supplementary figure 7. H&E staining of liver sections. Part II. Mice were injected with anti *Eef2* siRNA and sacrificed on specified days.

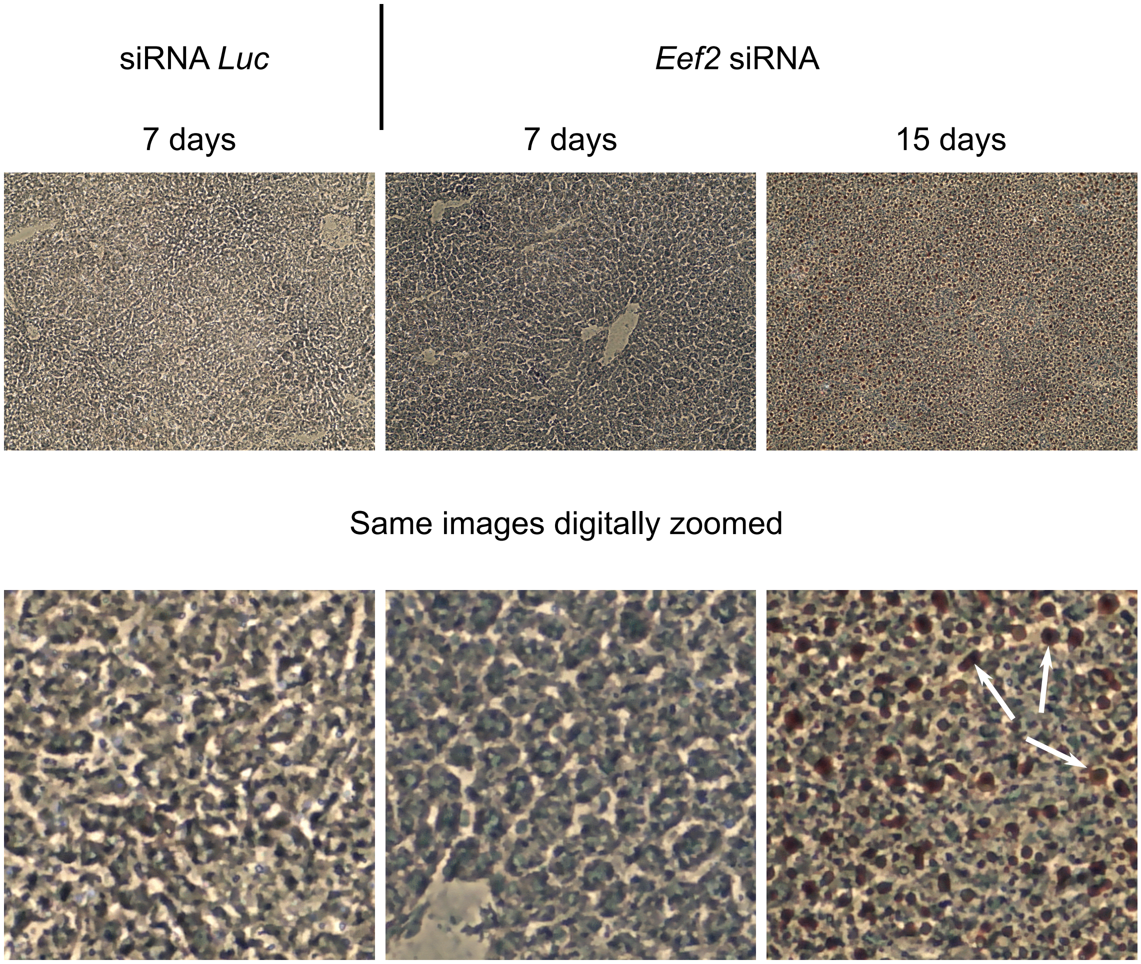

Supplementary figure 8. Oil Red staining of liver sections. Mice were injected with anti *Eef2* or anti *Luc* siRNA and sacrificed on specified days. Liver was preserved in a cryo medium, slides were stained for lipids with Oil Red stain. White arrows point to several representative lipid droplets.

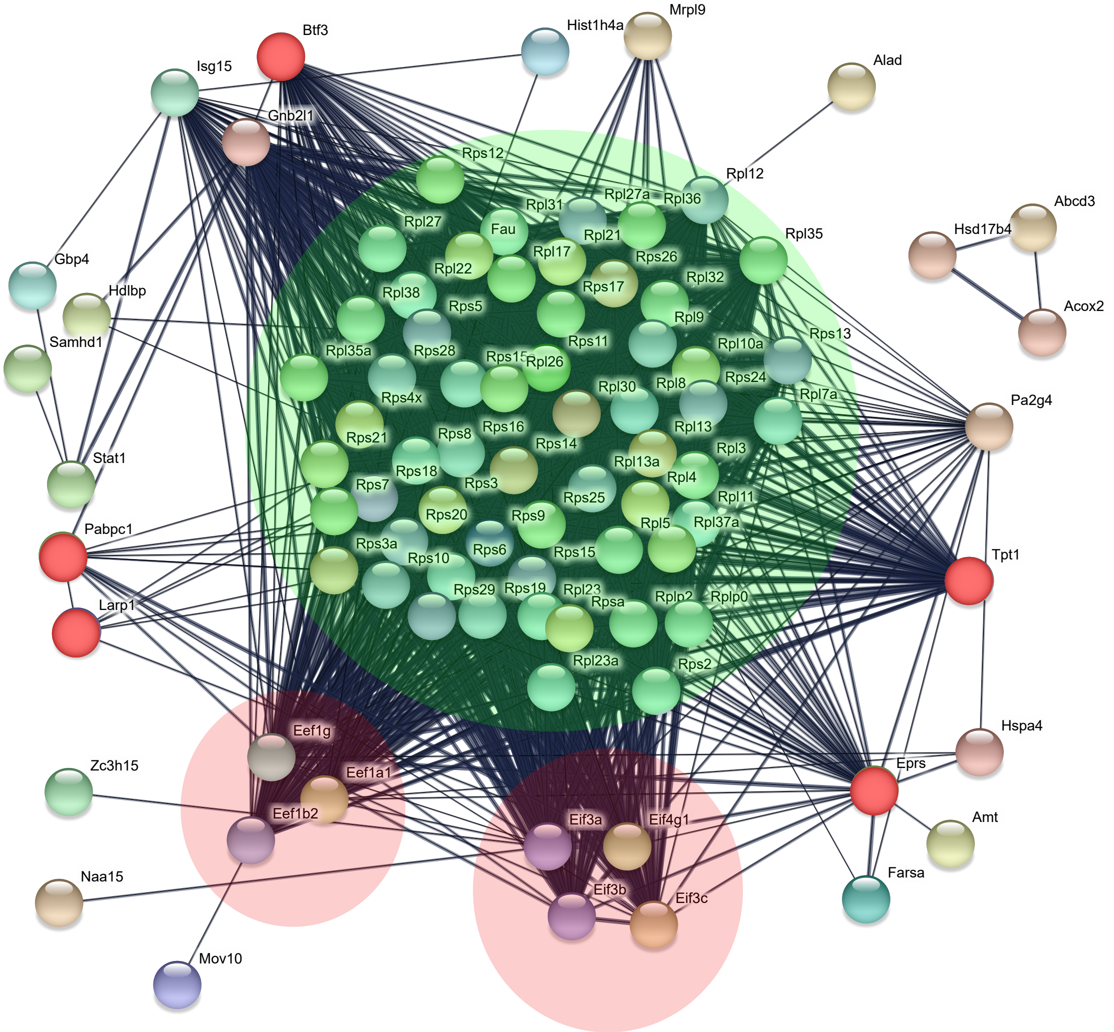

Supplementary figure 9. Proteins upregulated after 9 days of *Eef2* liver knockdown, mass spec data. Green cluster - cytoplasmic ribosome genes; red clusters - translation elingation and initiation factors; nodes highlighted in red corrspond to translation related proteins.

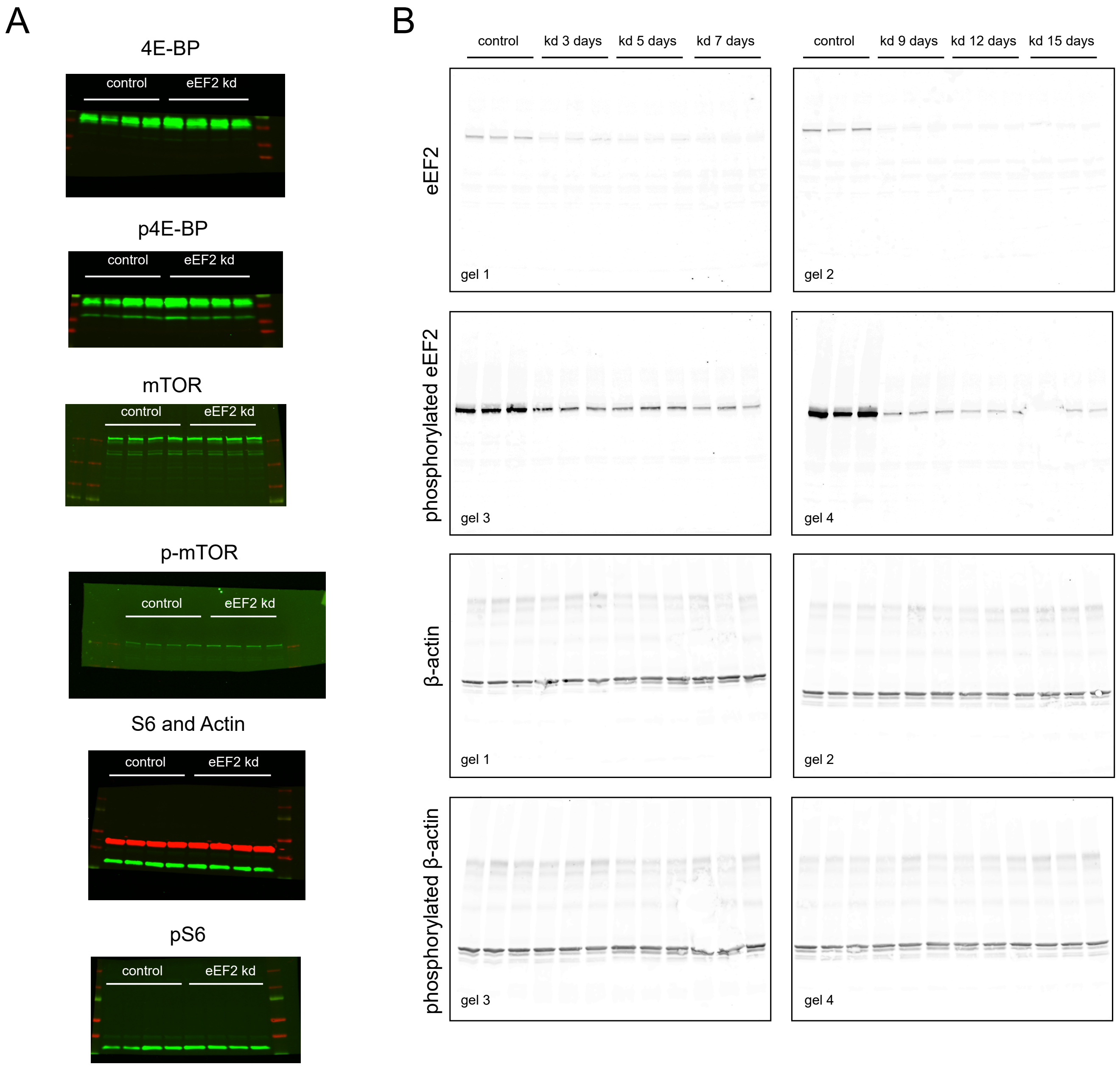

Supplementary figure 10. (**a**) Scans of the full size western blot membranes from in-vitro experiments (supplements Figure 1 in the main text). (**b**) Scans of the full size western blot membranes from in-vivo experiments (supplements Figure 2 in the main text). These gels show all individual replicates.

4EBP-1

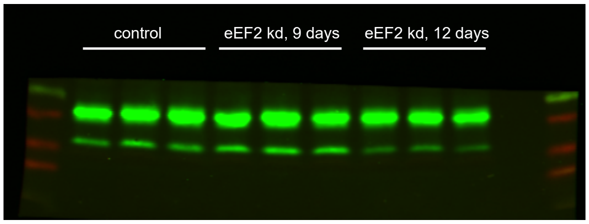

mTOR (green) and β-actin (red)

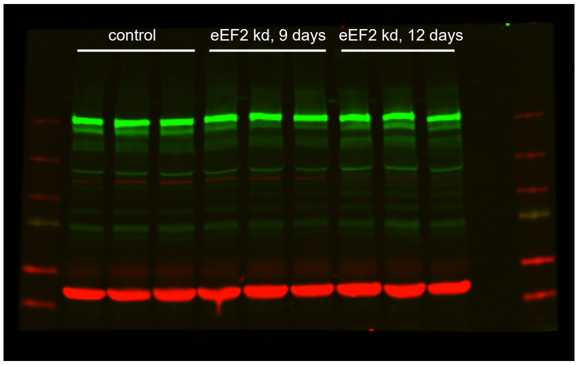

p-mTOR

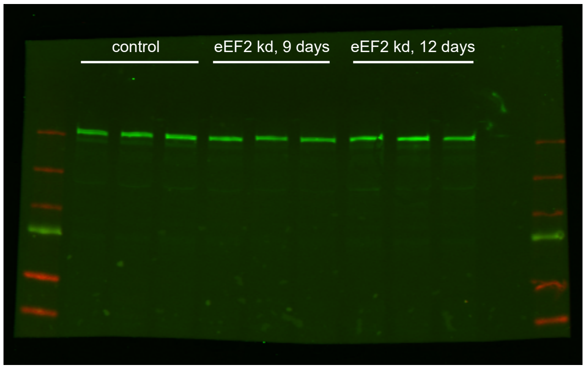

Supplementary figure 11. Scans of the full size western blot membranes from in-vivo experiments (supplements Figure 4E in the main text).

Supplementary Table 1. Serum biochemistry analysis of control and *Eef2* knockdown animals.

	Control mice	*Eef2* kd 7 days	*Eef2* kd 9 days	*Eef2* kd 12 days	*Eef2* kd 15 days	ANOVA p-value
Alkaline phosphatase (u/l)	79.7±5.0	60.3±5.8	61.7±10.1	42.0±3.6	56.0±2.0	0.0050*
AST (u/l)	55.0±10.4	88.7±61.2	131.0±21.0	161.3±61.6	496.3±133.9	0.0025*
ALT (u/l)	48.3±4.5	89.3±50.1	87.7±18.6	303.0±142.8	1319.3±279.5	<0.0001*
AST/ALT	1.13±0.15	0.95±0.12	1.53±0.36	0.57±0.13	0.37±0.04	0.0035*
Creatine kinase (u/l)	47.67±17.62	40.33±37.81	96.00±43.31	32.67±2.31	37.33±18.04	0.1057
Albumin (g/dl)	2.77±0.06	2.60±0.10	2.53±0.06	2.13±0.06	2.17±0.21	0.0024*
Total Bilirubin (mg/dl)	0.133±0.058	0.100±0.001	0.100±0.001	0.100±0.001	0.167±0.058	0.1705
Total Protein (g/dl)	4.93±0.21	4.67±0.23	4.33±0.21	3.67±0.15	3.60±0.26	0.0010*
Creatinine (mg/dl)	0.210±0.001	0.077±0.058	0.043±0.058	0.010±0.001	0.077±0.058	0.0494*
Cholesterol (mg/dl)	146.7±13.2	98.7±12.0	75.7±9.5	49.7±2.1	22.3±7.1	<0.0001*
Glucose (mg/dl)	277.7±17.6	242.3±58.0	216.0±37.6	255.0±60.0	253.3±37.2	0.5813
Phosphorus (mg/dl)	8.07±0.31	8.17±1.53	9.80±0.61	6.50±0.62	8.63±1.46	0.6248
Chloride (mmol/l)	107.3±0.6	107.7±1.2	110.3±0.6	109.0±1.7	107.7±0.6	0.4592
Potassium (mmol/l)	5.03±0.25	4.67±0.51	4.73±0.31	4.77±0.45	5.13±0.46	0.5818
Sodium (mmol/l)	143.7±0.6	144.0±1.7	146.7±1.2	144.3±1.2	144.7±1.2	0.0822
Triglycerides (mg/dl)	360.7±65.4	190.3±16.2	164.7±20.3	218.7±19.0	84.0±60.9	0.0033*
HDL Cholesterol (mg/dl)	82.7±6.4	64.3±6.1	52.3±3.8	38.7±0.6	13.7±6.5	<0.0001*
Bile Acids (umol/l)	6.6±0.7	13.1±8.9	11.9±13.8	21.8±4.9	25.3±18.0	0.2964

Supplementary Table 2. List of antibodies.

	Antibody	Manufacturer	Ref #	Dilution
Primary antibodies				
	β-Actin	Sigma	A1978	1:2000
	eEF2	Cell Signaling Technology	2332	1:1000
	p[T56]-eEF2	Cell Signaling Technology	2331	1:1000
	S6	Cell Signaling Technology	2217	1:1000
	p[S240/244]-S6	Cell Signaling Technology	2215	1:1000
	4E-BP1	Cell Signaling Technology	9644	1:1000
	p[T37/46]-4EBP1	Cell Signaling Technology	2855	1:1000
	mTOR	Cell Signaling Technology	2983	1:1000
	p[S2448]-mTOR	Cell Signaling Technology	2971	1:1000
Secondary antibodies				
	Goat anti-mouse IgG IRDye 680CW	LI-COR	926-68070	1:5000
	Goat anti-rabbit IgG IRDye 800CW	LI-COR	926-32211	1:5000

Supplementary Table 3. List of oligonucleotides.

	Name	Sequence (5' -> 3')
qPCR primers		
	Actb forward	GTGACGTTGACATCCGTAAAGA
	Actb reverse	GCCGGACTCATCGTACTCC
	Eef2 forward	ATCGCTGAACGCATCAAGC
	Eef2 reverse	TGCGCTGGAAGGTCTGGTA
siRNA		
	Eef2 sense	cAGCcAAGCUGAUCGAGAAdTsdT
	Eef2 antisense	UUCUCGAUcAGCUUGGCUGdTsdT
	Luc sense	cuuAcGcuGAGuAcuucGAdTsdT
	Luc antisense	uCGAAGuACUcAGCGuAAGdTsdT
Luciferase assays		
	forward	CCGCCGTAATACGACTCACTATAGG
	reverse	T(50)AACTTGTTTATTGCAGCTTATAATGG

N - standard nucleotide

n - nucleotide bearing 2'-O-methylation

dT - deoxythymidine

s - phosphorothioate bond

(50) - number of repeated residues
